# Scalable linkage-disequilibrium-based selective sweep detection: a performance guide

**DOI:** 10.1186/s13742-016-0114-9

**Published:** 2016-02-08

**Authors:** Nikolaos Alachiotis, Pavlos Pavlidis

**Affiliations:** 1 Department of Electrical and Computer Engineering, Carnegie Mellon University, 5000 Forbes Avenue, Pittsburgh, 15213 PA USA; 2 Institute of Molecular Biology and Biotechnology, Foundation for Research and Technology-Hellas, Crete, 70013 Greece

**Keywords:** Linkage disequilibrium, Omega statistic, High performance, OmegaPlus

## Abstract

**Background:**

Linkage disequilibrium is defined as the non-random associations of alleles at different loci, and it occurs when genotypes at the two loci depend on each other. The model of genetic hitchhiking predicts that strong positive selection affects the patterns of linkage disequilibrium around the site of a beneficial allele, resulting in specific motifs of correlation between neutral polymorphisms that surround the fixed beneficial allele. Increased levels of linkage disequilibrium are observed on the same side of a beneficial allele, and diminish between sites on different sides of a beneficial mutation. This specific pattern of linkage disequilibrium occurs more frequently when positive selection has acted on the population rather than under various neutral models. Thus, detecting such patterns could accurately reveal targets of positive selection along a recombining chromosome or a genome. Calculating linkage disequilibria in whole genomes is computationally expensive because allele correlations need to be evaluated for millions of pairs of sites. To analyze large datasets efficiently, algorithmic implementations used in modern population genetics need to exploit multiple cores of current workstations in a scalable way. However, population genomic datasets come in various types and shapes while typically showing SNP density heterogeneity, which makes the implementation of generally scalable parallel algorithms a challenging task.

**Findings:**

Here we present a series of four parallelization strategies targeting shared-memory systems for the computationally intensive problem of detecting genomic regions that have contributed to the past adaptation of the species, also referred to as regions that have undergone a selective sweep, based on linkage disequilibrium patterns. We provide a thorough performance evaluation of the proposed parallel algorithms for computing linkage disequilibrium, and outline the benefits of each approach. Furthermore, we compare the accuracy of our open-source sweep-detection software OmegaPlus, which implements all four parallelization strategies presented here, with a variety of neutrality tests.

**Conclusions:**

The computational demands of selective sweep detection algorithms depend greatly on the SNP density heterogeneity and the data representation. Choosing the right parallel algorithm for the analysis can lead to significant processing time reduction and major energy savings. However, determining which parallel algorithm will execute more efficiently on a specific processor architecture and number of available cores for a particular dataset is not straightforward.

**Electronic supplementary material:**

The online version of this article (doi:10.1186/s13742-016-0114-9) contains supplementary material, which is available to authorized users.

## Background

### Introduction

Positive selection is the tendency of beneficial traits to increase in prevalence (frequency) in a population and is the driving force behind adaptive evolution [1]. The selective sweep theory and the genetic hitchhiking model [2] can be used to identify traces of positive selection by detecting regions of reduced genetic variation (which are said to have undergone a selective sweep. A trait is considered beneficial when the following two requirements are met: i) it must increase the probability of survival and reproduction of the organism, and ii) it must be heritable, i.e., it can pass to the organism’s offspring. When these requirements are satisfied, population genetics theory predicts that the frequency of this trait will increase in the population. In modern genetic analyses, traits are the various forms of a gene (alleles). For instance, if we simplistically assume that the color of the eyes is controlled by a single gene only, then the various eye colors are determined by the alleles of this gene. Because a beneficial allele increases the fitness of the organism carrying it, its frequency will increase, and eventually all individuals of the population will possess it (the allele has reached fixation). Physically, alleles are located on chromosomal regions. Thus, on a chromosomal region, both a beneficial allele and alleles that do not affect fitness (neutral alleles) may be found. Given that entire chromosomal regions can potentially be inherited at once, beneficial alleles are often physically linked to several neutral ones, leading to the increase in frequency of linked neutral mutations near the site of the beneficial allele. Neutral alleles can also reach fixation, thus reducing the amount of polymorphism (number of single nucleotide polymorphisms, SNPs) near the beneficial mutation (by definition, fixation is monomorphic), and causing a selective sweep.

Selective sweep detection has theoretical significance and practical applications. For instance, it can shed light on the long-standing debate regarding the importance of adaptive and non-adaptive forces in shaping genetic polymorphisms [3]. Furthermore, it facilitates the detection of drug-resistant mutations in pathogens (e.g., HIV [4, 5]) that could reveal potential reasons for treatment failures [6] and lead to the design of more effective drug treatments.

Nowadays, the developments in DNA sequencing technologies, such as next-generation sequencing (NGS), are steadily contributing to an accelerating accumulation of molecular sequence data, because entire genomes can be rapidly and accurately sequenced in a cost-effective way [7]. Furthermore, the field of bioinformatics has many computationally demanding kernels^1^ that typically require prohibitively long processing times for the analysis of large-scale datasets available today. This issue has attracted the attention of the computer engineering community to such a great extent that the well-known Smith-Waterman pairwise sequence alignment algorithm [8] frequently serves as one of the test applications to demonstrate new engineering concepts in accelerator platforms. Additionally, several other compute- and/or memory-intensive bioinformatics kernels have been efficiently executed on emerging technologies such as multi-core central processing units (CPUs) [9–12], graphical processing units (GPUs) [9, 13], and field programmable gate arrays (FPGAs) [14–16]. The increased computational demands of the kernels used, in combination with the rapidly increasing pace at which genomes are sequenced today, generate an apparent challenge: how to devise new algorithmic solutions to effectively exploit new emerging technologies and eventually boost the capacity of modern processing architectures to keep up with the molecular data avalanche.

To this end, a software addition to the selective sweep detection computing landscape is the open-source program OmegaPlus [17], which has optimized parallel implementations for the analysis of genomic datasets of various shapes (dimensions), resulting from the varying number of sequences and SNPs. OmegaPlus captures linkage disequilibrium (LD) patterns of selective sweeps using the *ω* statistic [18], by analyzing SNPs in intra-species multiple sequence alignments (MSAs), essentially *n*×*m* data matrices that contain *n* DNA sequences of length *m* nucleotides each (also called alignment sites). The computational kernel of OmegaPlus is optimized for memory consumption, thus enabling the analysis of very large datasets on workstations with limited resources, such as personal computers. It relies on a computational approach that divides a dataset into a user-defined number of genomic regions and computes the *ω* statistic at the center of each region.

Before the release of OmegaPlus version 3.0.0 (January 2015), three parallelization alternatives for shared-memory systems were available: (i) a fine-grained algorithm that deploys all threads for the processing of a single genomic region, (ii) a coarse-grained algorithm that assigns a group of neighboring genomic regions to each thread, and (iii) a multi-grained algorithm [19] in which the master thread monitors the progress each thread has made with the assigned group of regions, and periodically directs all available threads (threads that have finished analyzing their initially assigned group using the coarse-grained algorithm) to assist, using the fine-grained approach, the slowest thread at that particular time. With the release of OmegaPlus v. 3.0.0, a new parallel algorithm is available, which implements a generic ‘offload-compute’ paradigm that organizes the LD calculations into chunks based solely on the available memory on the system, i.e., ignoring the genomic region borders, and carries out an iterative two-step procedure of LD calculations (the ‘offload’ step) followed by *ω* statistic calculations (the ‘compute’ step). All parallelization alternatives yield identical results, thus allowing performance to be improved without compromising the accuracy of the sweep detection method.

In this work, we present the aforementioned new generic parallel algorithm that relies on the offload-compute paradigm and conduct a thorough performance evaluation of all four available parallel alternatives. Furthermore, we assess the sensitivity and accuracy of OmegaPlus over other methods and tools that can be used for selective sweep detection via a series of simulated runs. From a performance standpoint, devising an efficient parallel algorithm for the *ω* statistic is challenging because of the variety of possible different input dataset types and shapes. For instance, a population genetics study may analyze either nucleotide data comprising a maximum of four possible states, A, C, G, and T, where one is ancestral and the remaining are derived, or assume the infinite site model [20], leading to the analysis of binary data where individuals can either carry an ancestral state (0) or a derived state (1). Previous experimental results revealed that LD calculations on DNA data require approximately 7–9 times more operations than on binary data, therefore significantly increasing the fraction of time required for LD calculations in comparison to *ω* calculations.

Furthermore, standard neutral simulated datasets, i.e., datasets of neutral SNPs from constant-size populations, typically show a more homogeneous SNP density than datasets with selection, because the area near selection is characterized by reduced amount of polymorphism as predicted by the selective sweep theory [2]. Such SNP imbalance between genomic regions translates to imbalance between individual per-thread execution times under the coarse-grained model. Additionally, the shape of the dataset (sample size and total number of SNPs) has a major effect on the computation-to-synchronization ratio of the available parallel algorithms, leading to significant performance deviations for the same number of threads, particularly as the number of threads increases.

The proposed generic algorithm distributes the computational load to so-called ‘compute groups’ without taking the borders of each genomic region into strict consideration. This leads to a small number of large monolithic blocks of computations that are organized into compute groups of equal size in terms of number of operations. Therefore, the proposed parallel algorithm achieves balanced load distribution to the threads regardless of the number of SNPs per candidate genomic region. Additionally, the generic offload-compute approach leads to reduced synchronization overhead in comparison with the previous parallel algorithms, which achieves high performance when the number of threads increases, even when small datasets are analyzed. The offload-compute paradigm implemented in OmegaPlus is inspired by typical hardware-based acceleration systems where frequent communication/synchronization between a host processor and the hardware accelerator is reduced (if not eliminated) and the computational blocks are ideally of the same size in order to achieve high system performance.

### Related work

Major advancements on modeling and statistical analysis for population genetics have been reported in the past 15 years [21–24]. However, high performance computing (HPC) remained largely underdeveloped before the NGS-driven data explosion, because of the limited amount of available population genetics data. More recently, the increasing number of sequenced genomes triggered the release of several software tools.

Pfeifer et al. [25] released PopGenome, an R package for population genetic analyses that can compute a wide range of statistics, infer parameters, and perform simulations. These can be applied to whole-genome SNP data and can exploit multiple cores for faster execution. A function is provided that calculates LD measurements for each pair of SNPs in a dataset, but a full implementation of the *ω* statistic is not available.

A variety of studies have focused on method development for selective sweep detection. Kim and Stephan [21] developed a composite maximum likelihood (ML) framework to detect selective sweeps based on empirical mutation frequencies of SNPs. This framework was adapted by Nielsen et al. [22] in the open-source software SweepFinder, which could analyze whole-genome datasets for a relatively small number of individuals. SweepFinder cannot exploit multiple cores and therefore has long execution times for genome-size analyses. Also, it cannot analyze alignments comprising more than 1,024 individuals. Pavlidis et al. [26] released SweeD, which uses the same maximum likelihood calculation core as the SweepFinder tool [22]. SweeD has a significantly faster sequential implementation and an efficient parallel algorithm. Also, SweeD can analyze alignments comprising tens of thousands of sequences. Additionally, a checkpointing mechanism allows to resume long-running analyses after system failures or queue timeouts on cluster systems.

Kim and Nielsen [18] described the LD signature of a selective sweep and introduced a ML framework and the *ω* statistic to detect selective sweeps based on LD. Pavlidis et al. [27] implemented the *ω* statistic and combined it with the approach proposed by Nielsen et al. [22] in a machine learning environment to improve the accuracy of the detection process. However, the complexity of the *ω* statistic implementation made the machine learning approach computationally infeasible on large datasets. Alachiotis et al. [17] introduced a dynamic programming (DP) algorithm for the efficient calculation of the *ω* statistic on large datasets and released the parallel software OmegaPlus, making the detection of selective sweeps on whole-genome MSAs with thousands of sequences feasible in a reasonable amount of time. The following section describes the *ω* statistic and presents the main algorithmic optimizations in OmegaPlus.

### The LD pattern of selective sweeps

LD is used to capture the non-random association of states between different SNPs. The level of LD can be quantified by a multitude of statistics. The most widely used measure of LD is the coefficient of linkage disequilibrium, D_LD_ [28]. Assuming the infinite site model (binary data), the D_LD_ coefficient is defined as D_LD_=*p*_*ij*_−*p*_*i*_*p*_*j*_, where *p*_*ij*_ is the frequency of haplotypes that have the state ‘1’ in both SNPs *i* and *j*, *p*_*i*_ is the frequency of haplotypes that have ‘1’ in SNP *i*, and *p*_*j*_ is the frequency of haplotypes that have ‘1’ in SNP *j*. Given that the range of D_LD_ depends on the frequencies of the states, normalized measures of LD are typically preferred. In this manuscript, we use the squared correlation coefficient between sites *i* and *j*, $r^{2}_{\textit {ij}} = \mathrm {D}_{\text {LD}}^{2} / (p_{i} (1 - p_{i}) p_{j} (1 - p_{j})) $, which always assumes values between 0 and 1. If state ‘1’ of SNP *i* is always associated with state ‘1’ of SNP *j*, then $r^{2}_{\textit {ij}}$ will be 1. On the other hand, if state ‘1’ of SNP *i* is associated with both states ‘1’ and ‘0’ of SNP *j*, then $r^{2}_{\textit {ij}}$ will be lower than 1.

**Fig. 1:**
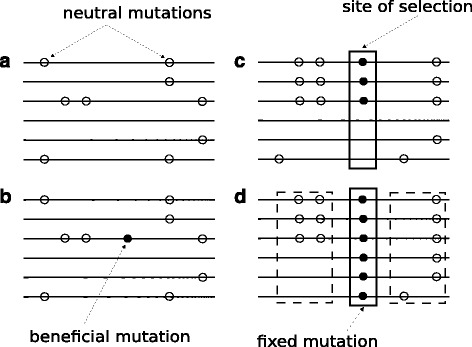
A selective sweep in a population. **a** Neutral mutations (open circles). **b** A beneficial mutation (filled circle) occurs. **c** The frequency of the beneficial mutation increases. **d** All individuals have the same mutation. The regions on the left and the right side of the selection site (dashed squares) comprise pairs of SNPs with high LD values. Pairs of SNPs on different sides have low LD

Figure 1 shows the generation of SNP patterns that can be used for selective sweep detection. It comprises four MSA snapshots of a population at different times. Open circles represent neutral mutations and filled circles indicate a beneficial mutation. Snapshot A is the oldest while snapshot D is the most recent. At some point in time, a beneficial mutation occurs in the population (snapshot B). Because this mutation is beneficial, the frequency of occurrence will increase (snapshot C), and eventually all individuals will have it (snapshot D). The LD between pairs of SNPs on the left- and right-hand side of the selective sweep is high, while LD between SNPs that are located on different sides of the beneficial mutation is low. A detailed explanation of the mechanisms that drive the generation of such SNP patterns near the site of selection is out of the scope of the present work. An excellent introduction into the evolutionary processes can be found in [29].

To compute the *ω* statistic, assume a total of *N* samples and a genomic region of *W* SNPs that is split into a left (*L*) and a right (*R*) subregion with *l* and *W*−*l* SNPs, respectively. We represent the states of the input data with *S*, where *S*_*DNA*_:{*A,C,G,T*} refers to DNA data and *S*_*BIN*_:{0,1} represents binary data. Initially, all $r_{s_{i}s_{j}}^{2}$ values are calculated as follows:

(1) $$ r_{s_{i}s_{j}}^{2} = \frac{\left({p_{s_{i}s_{j}}} -p_{s_{i}}p_{s_{j}}\right)^{2}}{p_{s_{i}}p_{s_{j}}\left(1-p_{s_{i}}\right)\left(1-p_{s_{j}}\right)},   $$

where *s*_*i*_,*s*_*j*_∈*S*, $p_{s_{i}}$ is the number of *s*_*i*_ states in SNP *i* divided by the total number of states *N*, $p_{s_{j}}$ is the number of *s*_*j*_ states in SNP *j* divided by the total number of states *N*, and $p_{s_{i}s_{j}}$ is the number of *s*_*i*_*s*_*j*_ pairs of states divided by the total number of pairs of states *N*. If *S*=*S*_*BIN*_, i.e., the input data are in binary format, then $r_{\textit {ij}}^{2}=r_{s_{i}s_{j}}^{2}$. For DNA input data, $r_{\textit {ij}}^{2}$ is calculated as follows, according to [30]:

(2) $$ r_{ij}^{2} = \frac{(v_{i}-1)(v_{j}-1)v_{ij}}{v_{i}v_{j}} \sum_{s_{i},s_{j}\in S}r_{s_{i}s_{j}}^{2},   $$

where *v*_*i*_ is the number of existing states in SNP *i* (*v*_*i*_≤4), *v*_*j*_ is the number of existing states in SNP *j* (*v*_*j*_≤4), and *v*_*ij*_ is the number of valid pairs of states (*s*_*i*_,*s*_*j*_∈*S*, i.e., not alignment gaps). The *ω* statistic is then computed as follows:

(3) $$ \omega = \frac{\left({l \choose 2} + {W-l \choose 2}\right)^{-1}\left(\sum_{i,j\in L}r_{ij}^{2} + \sum_{i,j\in R}r_{ij}^{2}\right)}{\left(l(W-l)\right)^{-1}\sum_{i\in L, j\in R}r_{ij}^{2}}.   $$

**Fig. 2:**
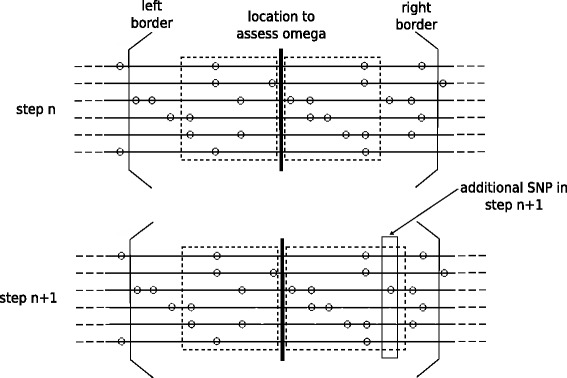
Two consecutive calculations of the *ω* statistic at the center (thick vertical line) of a subgenomic region, as defined by the left and right borders. The dashed squares show the SNPs that are included in each calculation step. Note the additional SNP in the right dashed square in step *n*+1

The area between the left and the right subregions is considered as the center of the selective sweep. The *ω* statistic quantifies the extent to which average LD is increased on each side of the selective sweep (numerator in Eq. 3) but not across the site of selection (denominator in Eq. 3). In subgenomic regions, i.e., candidate regions of limited length (usually some thousands of bases long), the *ω* statistic is computed at each interval between two SNPs as shown in Fig. 2. The length of the genomic region is based on a parameter provided by the user. The calculations aim to find the *l* that maximizes *ω* by evaluating Eq. 3 for all possible subregions that lie within the borders of the candidate region.

A grid of equidistant locations *L*_*i*_, 1<*i*<*D* is constructed based on the length of the input dataset. The parameter *D* is defined by the user. For each genomic region (centered at *L*_*i*_), the *ω* statistic is evaluated independently. OmegaPlus initially computes a lower triangular matrix *M* of the squared correlation coefficients $r_{\textit {ij}}^{2}$ between all SNPs *i* and *j*. Thereafter, a dynamic programming (DP) algorithm is used to calculate $\sum _{i\in L, j\in R}r_{\textit {ij}}^{2}$. Given a total of *W* SNPs in the genomic region, the matrix *M* of size *W*(*W*−1)/2 is updated as follows:

(4) $$ {}\begin{aligned} M_{i,j} = \left\{ \begin{array}{ll} 0 & \quad 1 \leq i \leq W, \ j = i \\ r_{ij}^{2} & \quad 2 \leq i \leq W, \ j = i - 1\\ M_{i,j+1} + M_{i-1, j} -\\ M_{i-1, j+1} + r_{ij}^{2} & \quad 3 \leq i < W, \ i-1 > j \geq 0.\\ \end{array}\right. \end{aligned}  $$

Thereafter, all $\sum _{i\in L, j\in R}r_{\textit {ij}}^{2}$, $\sum _{i,j\in L}r_{\textit {ij}}^{2}$, and $\sum _{i,j\in R}r_{\textit {ij}}^{2}$ values required by Eq. 3 are retrieved from *M*.

Typically, the number *D* of locations *L*_*i*_, 1<*i*<*D*, is in the order of thousands. This frequently leads to extended overlapping areas between neighboring genomic regions. To avoid redundant calculations in such cases, OmegaPlus uses a data-reuse optimization. Given a matrix *M*_*i*_ that corresponds to the genomic region *L*_*i*_, the matrix *M*_*i*+1_ is calculated in two steps. First, values from the lower *n* rows of *M*_*i*_ are copied to the higher *n* rows in *M*_*i*+1_, where *n* is the number of SNPs in the common region between genomic regions *L*_*i*_ and *L*_*i*+1_. Thereafter, the remaining rows in *M*_*i*+1_ are calculated. This optimization can achieve up to an order of magnitude faster overall execution, depending on the number of genomic regions and the extent of the overlaps [17].

## Findings

### Parallel algorithms for the ***ω*** statistic

#### Fine-grained algorithm (OmegaPlus-F)

**Fig. 3:**
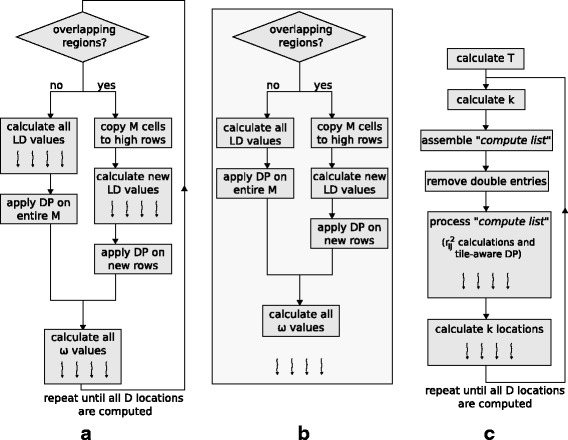
The algorithmic steps of three parallelization strategies in OmegaPlus. **a** The fine-grained approach to calculate one *ω* location; **b** the coarse-grained approach executed by each thread to analyze a genomic region; **c** the new parallel algorithm to compute a number *k* of *ω* locations. The wavy arrows indicate execution by multiple threads

The fine-grained parallel approach deploys all threads for the analysis of the same candidate genomic region, following the steps in Fig. 3a. Before the analysis of a new region, the master thread checks whether the data-reuse optimization can be applied. If the regions are not overlapping, the matrix *M* is fully calculated by parallel threads and filled with the new squared correlation coefficients. If there is overlap, the master thread copies the common values to the appropriate locations for the new region and only a part of *M* is calculated in parallel. Thereafter, the master thread applies the DP algorithm (Eq. 4) to update *M* with the summation values required by Eq. 3. The final step for the analysis of a region entails the parallel calculation of the *ω* statistic, with each thread computing *ω* independently for different *l* values (see Eq. 3).

A major issue with the performance of the straightforward fine-grained approach lies in the fact that all threads analyze a single genomic region: when the number of sequences is small (up to the order of thousands), the computation of squared correlation coefficients is relatively fast compared to the thread synchronization overhead. When the sample size increases, the computation-to-synchronization ratio improves because the calculation of the squared correlation coefficients requires more operations. Nevertheless, the frequent synchronization events and the small (because of the data-reuse optimization) number of squared correlation coefficients that need to be calculated for each genomic region do not allow for efficient parallel execution unless the sample size is in the order of tens of thousands. Consequently, the coarse- and multi-grained alternatives typically outperform the fine-grained approach.

#### Coarse-grained algorithm (OmegaPlus-C)

Unlike the fine-grained algorithm, the coarse-grained scheme (Fig. 3b) organizes the entire dataset into larger regions and assigns a different region to each thread, which carries out all *ω* statistic operations in that region sequentially. Each region comprises an equal number of candidate genomic regions, thus eliminating the need for synchronization during the analysis. Threads synchronize only once, to determine whether the analysis of all assigned regions is completed.

The coarse-grained approach benefits the most from the data-reuse optimization because all squared correlation coefficients are calculated by a single thread. However, the coarse-grained assignment of large regions to parallel threads suffers from imbalanced load distribution because, although each region consists of the same number of candidate genomic regions, each individual region that is scanned for selection contains a different number of SNPs.

#### Multi-grained algorithm (OmegaPlus-M)

The multi-grained algorithm attempts to alleviate the load imbalance in the coarse-grained approach via a more intelligent assignment of threads to regions. Initially, the algorithm is similar to the coarse-grained scheme because each thread is assigned a large region of equal size (number of candidate genomic regions) and carries out all required operations independently. When a thread finishes processing its initial region, the master thread, which has been monitoring the progress that each thread has made with the assigned region, dynamically decides which region the available thread must be reassigned to. Then, the selected region starts being analyzed by multiple threads following the fine-grained algorithm while the other regions continue being analyzed by one thread each. Depending on the overall progress in the regions, the master thread periodically reassigns threads to another region in order to achieve a balanced distribution of the remaining computational load at each particular time.

While the multi-grained scheme typically outperforms the coarse-grained algorithm, as more threads enter the fine-grained processing mode, the computational load per thread decreases and the synchronization overhead increases. Thus, the load distribution to the threads and the overall execution time for an analysis are still affected by the different number of SNPs in the regions.

### Generic parallelization (OmegaPlus-G)

#### Data layout

**Fig. 4:**
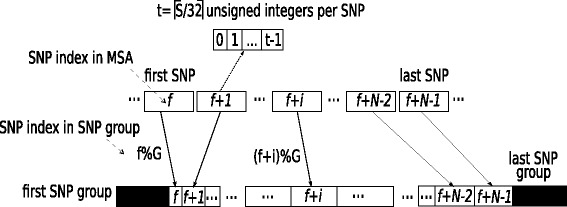
All *N* SNPs in a subgenomic region are mapped to distinct locations in SNP groups of size *G*. Assuming 32-bit unsigned integers and *S* sequences, each SNP occupies *t* integers (ceiling of *S* sequences divided by 32). Each SNP is placed in a SNP group based on it’s location *i* in the subgenomic region, where *f* is the index of the first SNP in the subgenomic region of *N* SNPs

Initially, the input alignment is stored in an array of 32-bit unsigned integers by grouping 32 elements at an alignment site (0, 1 for binary data, and A, C, G, T for DNA data) in each unsigned integer and placing SNPs contiguously in the array, as shown in Fig. 4. In addition to reducing memory footprint, such a compact representation of SNPs facilitates the population count operation required to calculate the squared correlation coefficient between SNPs. Thereafter, the SNPs are organized into SNP groups of size *G*, where *G* is a granularity factor that affects the ratio of computation to synchronization among the threads. Figure 4 also illustrates the arrangement of SNPs into groups for a genomic region that comprises *N* SNPs. The value of *G* is a constant that was set to 128, based on an experimental evaluation of the effect of *G* on the total execution time. In a series of runs with *G* values ranging from 8 to 1,024, a significant performance degradation was observed for values smaller or larger than the chosen *G*. Small *G* values reduce the number of computations between synchronization events and increase the retrieval time of the computed values during the final *ω* calculations. On the other hand, large *G* values lead to execution time imbalance among the threads because of very coarse-grained load distribution, and several redundant computations between very distant SNPs. SNPs are placed in groups based on their alignment site index to facilitate the application of the proposed algorithm directly on the data layout of the standard OmegaPlus implementation.

#### Parallel algorithm

The underlying idea of the proposed algorithm is to maximize the number of calculations in the parallel sections of the code while each thread performs computations on fractions of genomic regions. The algorithmic steps are outlined in Fig. 3c. Initially, a total of *T* available SNP groups is assumed. The value of *T* is calculated from a user-defined parameter that provides an upper limit for the memory overhead induced by the proposed algorithm. As previously mentioned, the SNP-group size *G* is fixed to 128 for performance purposes. Consequently, each genomic region frequently extends to several SNP groups. Therefore, for a given number *T* of SNP groups, the calculation of the *ω* statistic is possible at only a limited number of locations *L*_*i*_, 1<*i*<*k*≪*D*, where *D* is defined by the user. The borders of the respective *k* genomic regions are then analyzed to assemble a ‘compute list’. Each item in the list is a ‘compute group’. Because of possible overlaps between neighboring genomic regions, a data reduction operation on the compute list eliminates double entries that would otherwise have led to redundant computations.

**Fig. 5:**
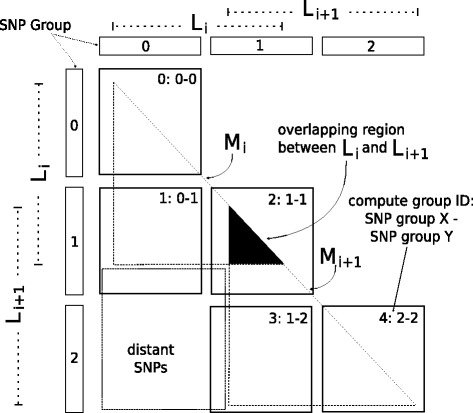
Two neighboring subgenomic regions *L*_*i*_ and *L*_*i*+1_ organized into three SNP groups and the respective compute list that comprises five compute groups

A compute group operates on a distinct pair of SNP groups *x* and *y* that belong to the same genomic region, as illustrated by the square tiles in Fig. 5. Initially, a kernel function that calculates the squared correlation coefficient $r_{\textit {ij}}^{2}$ for a pair of SNPs *i* and *j* with *i*∈*x* and *j*∈*y* is invoked *G*^2^ times. If *x*=*y*, the kernel function is invoked *G*^2^/2 times (compute groups 0, 2, and 4 in Fig. 5) to avoid redundant calculations. When the $r_{\textit {ij}}^{2}$ calculations are finished, a tile-aware version of the DP algorithm (Eq. 4) is applied. A data dependency resulting from the DP algorithm imposes a corresponding tile dependency. Therefore, only the compute groups that belong to the same diagonal can be calculated in parallel, and each diagonal can be processed only after the one directly above is computed. In Fig. 5 for instance, compute groups 0, 2, and 4 represent the first diagonal that can be calculated in parallel. Thereafter, the next diagonal, comprising compute groups 1 and 3, can be calculated in parallel.

The final stage of the proposed parallel algorithm entails the *ω* statistic calculation at *k* locations *L*_*i*_, 1<*i*<*k*, where *k* is calculated based on the available number of SNP groups *T*. For this, we modified the standard OmegaPlus function that computes the *ω* statistic at each location *L*_*i*_ to retrieve the $\sum _{i\in L, j\in R}r_{\textit {ij}}^{2}$, $\sum _{i,j\in L}r_{\textit {ij}}^{2}$, and $\sum _{i,j\in R}r_{\textit {ij}}^{2}$ values from the tiled output data. Thereafter, all *L*_*i*_ locations are distributed to so-called omega queues based on the total number of SNPs in each queue. When all *L*_*i*_ locations are inserted into an omega queue, each queue comprises approximately the same number of SNPs. Therefore, by maintaining a queue per thread we achieve improved load balance for the final *ω* statistic calculations.

**Fig. 6:**
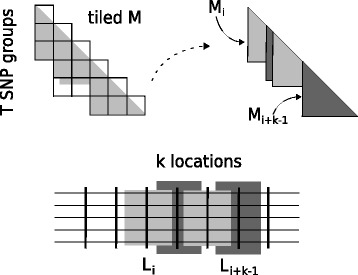
Example of the tiled LD data for an arbitrary iteration of the generic algorithm along with the corresponding per-region *M* matrices and the *ω* locations that can be calculated. Here, the number of SNP groups is *T*=6, the pairwise combination of which leads to the tiled matrix *M* that comprises 16 tiles (missing tiles correspond to distant SNPs, as determined by the −*maxwin* user-defined argument, and therefore are not computed). The corresponding number of *ω* locations that can be calculated in this iteration is *k*=4, i.e., *L*_*i*_ to *L*_*i*+*k*−1_

The above-mentioned steps are repeated until all locations *L*_*i*_, 1<*i*<*D*, are analyzed. Figure 6 illustrates an example of output tiled data for an arbitrary iteration of the algorithm, the corresponding *M* matrices, and the *ω* locations that can be calculated. Each iteration assumes a different set of *T* SNP groups (*T*=6 in Fig. 6) and computes the next *k* locations in the input dataset (*k*=4 in Fig. 6). Note that, although *T* (number of SNP groups) is constant among iterations of the algorithm (it relies only on the available memory resources on the workstation), the actual number *k* of *L*_*i*_ locations that can be calculated might vary because of variances in the SNP density among genomic regions. As already mentioned, each genomic region extends to several SNP groups. Therefore, SNP regions with an increased number of SNPs, for instance, will occupy a proportionally increased number of SNP groups, leading to a smaller number *k* of *L*_*i*_ locations that can be computed based on the available *T* SNP groups.

### Implementation and usage

The generic algorithm is implemented in the OmegaPlus Linux release version 3.0.0 (available for download at [31] and [32]) using the OpenMP application programming interface (API) for parallel programming. All previous parallel implementations used the POSIX standard for threads, a low-level API that was required to implement the multi-grained algorithm that exploits both the fine- and the coarse-grained alternatives. However, the simplicity of the code for the generic algorithm allowed deployment of the more portable OpenMP API because the computational kernel is implemented via two main *for* loops, one to traverse all tile diagonals in the tiled matrix M to process the compute list and one to compute all omega queues.

**Fig. 7:**
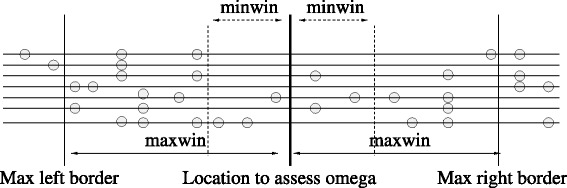
The roles of −*minwin* and −*maxwin*. The user defines the length of *minwin* (number of bases). This is the minimum region on the left or on the right of a given location that a sweep might affect. For example, for −*minwin* 1000, the SNPs located at least at a distance < 1,000 bp (1,000 bp on the right, and 1,000 bp on the left) from each grid point will contribute to the calculation of *ω* value. In this figure, the first *ω* value that will be calculated (its position is denoted as ‘location to assess omega’) will include three SNP positions on the left and three SNP positions on the right. Subsequently, OmegaPlus will gradually include in the calculations all SNPs on the left and right subregions one after the other until the ‘Max left’ and ‘Max right’ borders are reached. ‘Max left border’ and ‘Max right border’ are defined by the *maxwin* flag. For this position, the highest *ω* value is reported

To generate the OmegaPlus-G executable that implements the generic parallel algorithm, one can use the respective makefile (Makefile.OPENMP.GENERIC.gcc). A typical analysis requires at least five input arguments: (i) a name for the run (*-name*), (ii) an input file (*-input*), (iii) the number of *ω* locations (*-grid*), and (iv) a minimum (*-minwin*) and a maximum (*-maxwin*) border value to specify the width of each candidate genomic region. The generic algorithm requires an additional argument that is provided via the *-memLimit* command line flag and represents an upper bound for the memory overhead. The roles of the minimum and maximum border values for the processing of genomic regions are illustrated in Fig. 7.

Regardless of the chosen OmegaPlus algorithm, all runs generate a report that contains locations in the genome (at the center of each evaluated region) and the corresponding *ω* scores. To assess whether the reported OmegaPlus scores are indicative of a selective sweep, a threshold calculation step is required to determine the critical OmegaPlus value. All regions that are scored higher than the critical value are candidates for a selective sweep.

Threshold calculation is based on the definition of *p*-value: the probability of observing equal or higher value than the maximum observed OmegaPlus value given that the null hypothesis is true. The null hypothesis is represented by a neutral model that incorporates an appropriate demographic scenario. In other words, if the demographic model is known, neutral datasets are generated via simulations incorporating the demographic model. For each simulated dataset, the maximum OmegaPlus value is calculated, and the threshold value is defined as the 95th percentile of all maxima. In practice, however, this approach requires two problems to be resolved: i) the demographic model is usually unknown, so it needs to be estimated, and ii) if the region under study and the recombination rate are large, simulating neutral datasets becomes challenging. Several approaches exist for the estimation of the demographic model (e.g., the approximate Bayesian computation, ABC; [33]) and the simulation of neutral datasets (e.g., Hudson’s ms [34] and msms [35] for full coalescent models, or MaCS [35] for approximate coalescent models). As an alternative to simulating neutral datasets, which might be computationally expensive, one may assume that the vast majority of the dataset under investigation is neutral. This allows to estimate the threshold value by considering the 95th percentile of the OmegaPlus values calculated from the analysis of the specific dataset, an approach that is usually followed for the analysis of whole-genome datasets.

### Performance

#### Experimental setup

To assess the performance of the parallel algorithms for LD-based selective sweep detection, we use the open-source software CoMuS (Coalescent of Multiple Species; [36]) to generate a series of datasets in a variety of shapes, with sample size m and number of sites k, where m = (100, 1,000, 10,000, 50,000) and k = (10,000, 50,000, 100,000). In contrast to Hudson’s ms [34], CoMuS implements the finite site model, therefore it generates DNA sequence data. The sample size varies from 100 to 50,000 because our goal is to test the new parallel algorithm in OmegaPlus for relatively small datasets that are currently widely used, and for very large datasets that will become available in the future. For the number of sites, we use values from 10,000 to 100,000. Such a number of sites corresponds to a genomic region, i.e., it is smaller than chromosomal sizes. Simulating longer DNA sequences becomes prohibitively expensive both in terms of computational time and memory requirements, because of the large amount of recombination.

We use a workstation with four Intel Xeon X7560 8-core Nehalem-EX processors running at 2.26 GHz and 128 gigabytes of main memory, as a test platform. All parallel OmegaPlus algorithms are evaluated for scalability with runs of 4, 8, 16, and 32 threads. For all runs, the *-grid*, *-minwin*, and *-maxwin* arguments are set to 5,000, 1,000, and 20,000, respectively. Therefore, all analyses compute the *ω* statistic at 5,000 locations along the input dataset with the minimum and the maximum sizes of the candidate genomic regions set to 1,000 and 20,000 alignment sites, respectively.

Typically, the number of candidates for selection genomic regions that should be evaluated in an input dataset should be proportional to the width of the dataset, i.e., the number of alignment sites. Although this is generally valid for real-world analyses, it would create a bias in our scalability evaluation experiments. Changing the *-grid* argument according to the number of sites k in the input datasets will maintain the number of SNPs in each region and the overlap between neighboring regions approximately constant, while the shape of the datasets will vary significantly. Therefore, the runtime comparisons will not capture the effect of the size of candidate regions and the extent of the overlaps.

#### Simulated datasets

**Fig. 8:**
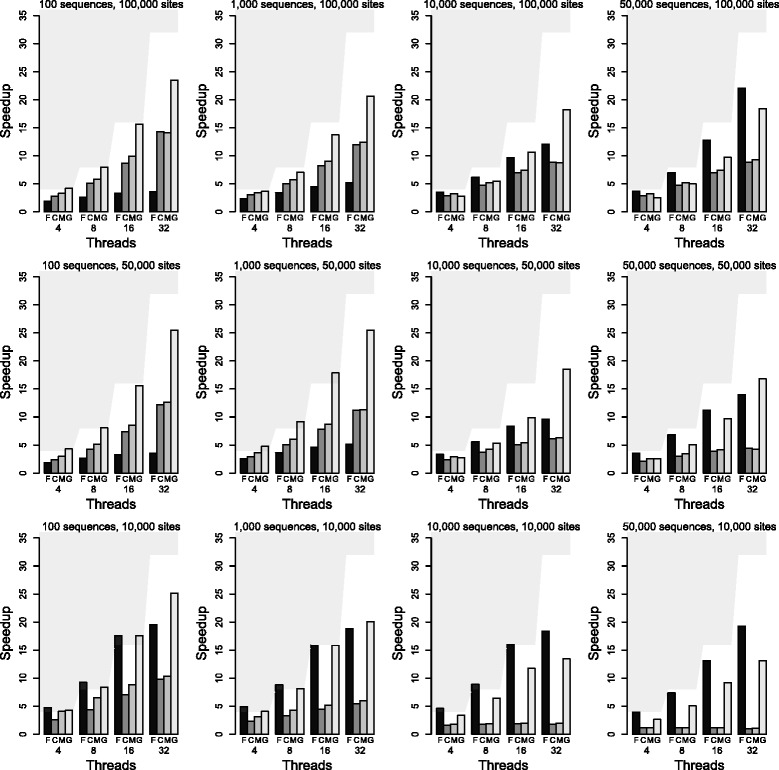
Speedups for the analysis of DNA datasets in different shapes. The sequential OmegaPlus implementation is take as reference for the calculation of the speedups; F, C, M, and G correspond to the fine-grained, coarse-grained, multi-grained, and generic parallel algorithms, respectively

Initially, we evaluate the scalability of the parallel algorithms on simulated datasets. Figure 8 shows the observed speedups.

As can be observed in the plots, the coarse- and multi-grained approaches show poor performance for limited number of sites (k = 10,000) achieving maximum speedups of 8.8X and 9.3X on 32 cores, respectively. This results from the small number of SNPs per group of regions, which decreases as the number of threads increases, leading to unfavorable computation-to-synchronization ratios. The fine-grained and the generic algorithms achieve speedups of up to 19.3X and 22.6X on 32 cores, respectively. Consequently, the fine-grained and the generic algorithms outperform the coarse- and multi-grained alternatives, showing even superlinear speedups for small sample sizes (up to 1,000) and numbers of threads (up to 8) because of the efficient exploitation of the cache hierarchy. For instance, when processing the DNA dataset with 100 sequences and 10,000 alignment sites (approximately 5,000 SNPs; bottom left of the figure), OmegaPlus allocates 64 bytes per SNP (4 unsigned integers per DNA state), allowing the loading of nearly 512 SNPs in L1 cache (32 kilobytes). Because all threads in the fine-grained model analyze the same region and the number of regions is large (*-grid 5000*), the majority of SNPs that will be required for a large number of consecutive overlapping neighboring regions already reside in L1 cache. The same cache effect is not observed for the coarse-grained approach because each thread starts analyzing distant regions. Although each thread’s next region contains several SNPs that already reside in L1 cache from the analysis of the previous region, the actual number of remaining regions that can benefit from SNPs in L1 cache is reduced proportionally to the number of parallel threads. Furthermore, owing to extensive overlaps and the small number of SNPs, a large number of redundant LD calculations may occur as a certain number of overlapping regions (at the borders of the region groups) are analyzed by different threads. The multi-grained approach outperforms the coarse-grained alternative and benefits from the cache effect because threads dynamically switch to the fine-grained model during processing. Finally, the generic approach requires only 16 kilobytes of L1 cache per compute group, leading to the efficient use of the cache hierarchy during the analysis of the compute list because the 2*G* SNPs (*G* is the granularity factor currently set to 128) per compute group will reside in L1 cache and be used in *G*^2^ pairwise LD calculations. However, as the sample size increases the memory requirements per SNP increase, the benefits from the cache hierarchy are reduced (towards the top right in Fig. 8), and the fine-grained approach outperforms the generic algorithm as a result of a favorable computation-to-synchronization ratio during the LD calculations.

Tables 1 and 2 provide execution times for DNA and binary datasets, respectively. Note that all datasets generated by CoMuS contain DNA data in FASTA format. To evaluate the performance of the parallel algorithms when binary SNPs are analyzed, we use the *-binary* OmegaPlus input argument, which enforces a deduction of the alignment to a binary representation before the analysis. The execution time reduction for the same dataset can be considerable as the dataset size increases. For instance, the sequential analysis of the largest simulated dataset in our experiments (m = 50,000 and k = 100,000) finished 7.7X faster by activating binary deduction, reducing the total analysis time from 24.6 hours to approximately 3 hours. The maximum *ω* value calculated based on DNA data was 1.281763 at location 99,973.78, whereas the respective values based on binary data were 1.235877 and 99,993.78.

**Table 1:** Execution times (in seconds) for the analysis of DNA datasets in different shapes

Sequences	100				1,000				10,000				50,000		
Sites	10,000	50,000	100,000		10,000	50,000	100,000		10,000	50,000	100,000		10,000	50,000	100,000
OP (1)	158	2,117	2,677		301	3,233	3,883		846	10,486	17,547		2,927	33,592	88,528
F (4)	37	1,143	1,381		61	1,256	1,687		184	3,160	5,011		744	9,438	23,798
C (4)	68	893	949		129	1,096	1,288		520	4,346	6,067		2,576	16,239	30,501
M (4)	43	710	811		95	896	1,135		479	3,626	5,358		2,518	13,172	27,399
G (4)	41	491	636		74	674	1,052		249	3,832	6,280		1,105	13,070	34,983
F (8)	19	790	1,008		34	898	1,136		95	1,880	2,843		394	4,924	12,610
C (8)	40	493	524		91	640	771		474	2,814	3,687		2,508	11,039	18,558
M (8)	27	411	463		70	539	676		450	2,467	3,351		2,458	9,623	17,063
G (8)	21	263	336		37	352	551		132	1,956	3,205		580	6,653	17,713
F (16)	10	649	809		19	700	867		53	1,255	1,812		223	3,003	6,922
C (16)	25	288	308		67	413	473		445	2,063	2,522		2,447	8,537	12,701
M (16)	20	247	270		58	370	428		432	1,953	2,355		2,429	8,114	11,882
G (16)	10	136	171		19	181	283		72	1,063	1,651		319	3,457	9,125
F (32)	9	599	745		16	632	748		46	1,094	1,450		152	2,405	4,009
C (32)	18	174	187		55	289	324		479	1,705	1,985		3,014	7,543	10,041
M (32)	17	168	190		50	286	313		440	1,674	1,999		2,782	7,897	9,566
G (32)	7	83	114		15	127	188		63	567	962		223	2,001	4,814

OP indicates the sequential OmegaPlus implementation; F, C, M, and G indicate the fine-grained, coarse-grained, multi-grained, and generic parallel algorithms, respectively. The number in parentheses is the number of threads

**Table 2:** Execution times (in seconds) for the analysis of binary datasets in different shapes

Sequences	100				1,000				10,000				50,000		
Sites	10,000	50,000	100,000		10,000	50,000	100,000		10,000	50,000	100,000		10,000	50,000	100,000
OP (1)	151	2,024	2,467		241	2,653	2,535		438	3,442	4,433		777	5,636	11,485
F (4)	39	1,238	1,480		67	1,313	1,569		135	1,664	2,009		252	2,364	4,380
C (4)	64	1,017	1,052		114	1,015	991		195	1,265	1,528		469	2,627	4,360
M (4)	40	701	861		69	796	889		151	1,099	1,398		401	2,205	4,008
G (4)	40	521	624		65	545	652		115	924	1,249		250	1,975	4,395
F (8)	19	795	1,011		32	838	1,002		53	1,012	1,214		134	1,438	2,684
C (8)	36	464	488		64	478	487		132	709	869		373	1,701	2,857
M (8)	23	379	423		41	408	445		105	638	813		335	1,483	2,681
G (8)	20	254	312		32	274	328		59	481	679		144	1,091	2,570
F (16)	10	652	808		17	675	828		33	834	975		97	1,250	2,250
C (16)	20	258	264		39	266	280		95	463	585		320	1,255	2,109
M (16)	16	222	243		30	233	250		84	429	549		306	1,198	2,021
G (16)	10	128	157		16	140	169		35	275	411		97	685	1,664
F (32)	9	598	699		15	623	743		31	764	860		84	1,066	1,919
C (32)	14	147	166		24	158	185		76	338	446		325	1,193	1,990
M (32)	16	137	165		22	161	173		77	337	446		313	1,133	2,064
G (32)	7	86	95		11	91	104		26	193	317		85	504	1,229

OP indicates the sequential OmegaPlus implementation; F, C, M, and G indicate the fine-grained, coarse-grained, multi-grained, and generic parallel algorithms, respectively. The number in parentheses is the number of threads

For moderate numbers of alignment sites such as k = 50,000 or k = 100,000, the generic parallel approach achieves up to 25.5X speedup on 32 cores whereas the fine-, coarse-, and multi-grained alternatives deliver maximum speedups of 22X, 16.8X, and 16.5X, respectively. Overall, the generic algorithm shows consistent performance on various different numbers of cores and is robust to changes in the dataset shape. Furthermore, because of the compute-list-based calculation of a significantly larger number of LD values than the other approaches in a single parallel region, the generic algorithm achieves good performance even when many cores are used to analyze datasets with limited sample size and number of SNPs.

#### Human Chromosome 1 from the 1000 genomes project

In addition to simulated datasets, we also evaluate the parallel algorithms for the scan of a very large real-world dataset, the Chromosome 1 of the human genome, available for download from the 1000 Genomes Project Consortium ([37], last accessed January 9, 2015). This dataset is in VCF format, which is widely used in next-generation sequencing projects, and contains the genetic variation from 2,504 humans, i.e., the sample size to be analyzed is 5,008 as a result of diploidy. The size of the input file is 62 gigabytes and comprises 6,195,844 SNPs. Similarly to previous performance comparisons, we conduct a performance evaluation of the parallel algorithms with runs using 4, 8, 16, and 32 threads, analyzing the input data from the VCF file as is, i.e., nucleotide data, as well as binary data, by activating the binary deduction optimization.

**Fig. 9:**
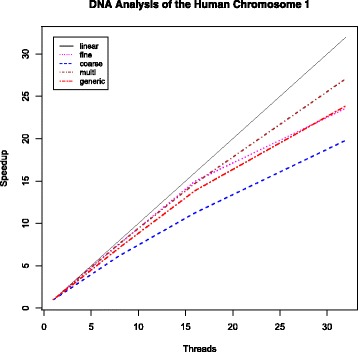
Observed speedups for the analysis of the human Chromosome 1 based on LD computations on nucleotide SNP data

**Fig. 10:**
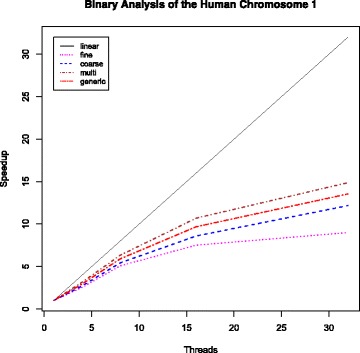
Observed speedups for the analysis of the human Chromosome 1 based on LD computations on binary SNP data

All runs compute the *ω* statistic at the center of 10,000 candidate regions (*-grid*) of minimum and maximum sizes of 1,000 (*-minwin*) and 200,000 (*-maxwin*) sites, respectively. Table 3 contains execution times and Figs. 9 and 10 illustrate the respective speedups. Given the considerable size of the input file (62 gigabytes), and so as to conduct an accurate evaluation of the parallel algorithms, the time required to load the file from the disk to main memory is not included in the execution times and the calculation of the respective speedups. All parallel OmegaPlus implementations use a single thread to load the input file to main memory before the analysis, a task that required between 80 and 85 min for all runs. However, the time required for the conversion of the nucleotide SNPs to binary data (carried out by a single thread as well) is included. This conversion required 24 min.

**Table 3:** Execution times (in seconds) for the analysis of the human Chromosome 1 (1000 Genomes project) conducting calculations based on DNA and binary data

Algorithm (threads)	DNA	Binary
OP (1)	417,161	44,195
F (4)	108,850	14,057
C (4)	129,712	15,266
M (4)	107,326	12,667
G (4)	115,653	13,474
F (8)	55,025	8,712
C (8)	67,371	8,142
M (8)	54,663	6,992
G (8)	58,341	7,492
F (16)	27,853	5,884
C (16)	37,091	5,145
M (16)	28,316	4,135
G (16)	30,089	4,567
F (32)	17,683	4,911
C (32)	21,043	3,630
M (32)	15,395	2,971
G (32)	17,469	3,262

OP indicates the sequential OmegaPlus implementation; F, C, M, and G indicate the fine-grained, coarse-grained, multi-grained, and generic parallel algorithms, respectively

As expected, because of the increased sample size and number of SNPs, all parallel algorithms show comparable performance and scale well as the number of threads increases (Fig. 9). The coarse-grained approach is the most prone to SNP density imbalance among regions, which reflects the worse performance compared with the rest of the algorithms. The large sample size allows highly favorable computation-to-synchronization ratio during the LD calculations of the fine-grained algorithm, whereas the multi-grained parallelization, which has the dynamic switching mechanism between the fine-grained and coarse-grained approaches, achieves the best performance because it benefits from both the efficient fine-grained LD calculations and the balanced workload per thread. Finally, because of the large number of SNPs in the dataset and the significantly wide candidate regions, the generic algorithm requires multiple offload-compute iterations, while each iteration can compute only a limited number of *ω* locations, thus leading to slightly worse performance than the multi-grained approach while outperforming the fine-grained approach when 32 threads are used.

The binary deduction optimization allows between 7X and 9.4X faster execution, including the time required to convert the DNA representation to binary (24 min). However, all parallel algorithms show poor performance as the number of threads increases (Fig. 10) because of the significantly reduced computational load per pairwise LD calculation. For binary analyses, the multi-grained and the generic algorithms are typically faster than the fine- and coarse-grained approaches because of the hybrid parallelization approaches used, which allow them to benefit from both the coarse-grained execution and the balanced computational load distribution to the threads, achieving favorable computation-to-synchronization ratios and similar execution times per thread.

**Fig. 11:**
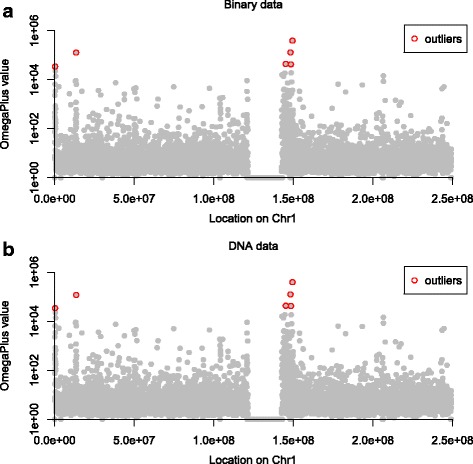
Illustration of the *ω* values reported by OmegaPlus for the analysis of the human Chromosome 1. **a** SNPs interpreted as nucleotide data (processed as is from the VCF file); **b** SNPs represented as binary vectors (*-binary* option). In both plots, the 10 highest *ω* values are highlighted. The DNA analysis took between 7 and 9.4 times longer than the binary analysis

Converting each SNP from DNA to binary is a non-deterministic process that leads to loss of information, because all nucleotide SNPs are now represented by binary vectors. Figure 11 illustrates the *ω* values that were computed based on LD values calculated from nucleotide SNPs (Fig. 11a) and binary data (Fig. 11b). The plots appear almost identical and there are no obvious differences in the *ω* statistic values calculated by the two approaches throughout the dataset (OmegaPlus output reports available as Additional file 1). In both plots, the ten highest (i.e., 0.1 %) *ω* values are highlighted (red circles). Both analyses detected the maximum *ω* value at position 149,445,280, and the leftmost and rightmost SNPs in the region that showed the maximum *ω* were at positions 149,433,322 and 149,448,635, respectively. When the SNP data were analyzed as DNA, the maximum *ω* value was 396,171.53, whereas when the SNPs were converted to binary the maximum *ω* value was 391,617.78. Note, however, that the sequential DNA analysis required 117.2 hours whereas the binary analysis finished in just 13.6 hours.

**Fig. 12:**
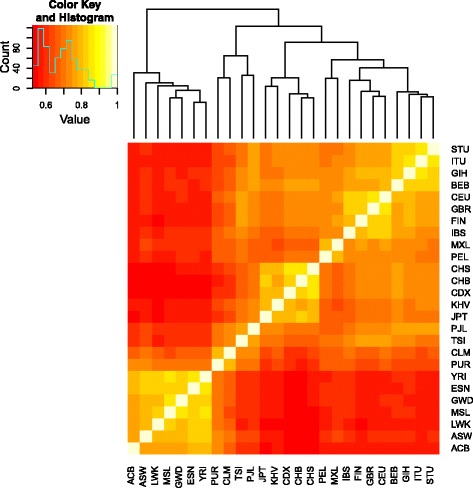
A heatmap of the 26 populations in the 1000 Genomes dataset for Chromosome 1. The similarity matrix was calculated using the Spearman correlation coefficient between the OmegaPlus scores of different populations. Light yellow indicates higher similarity (higher Spearman correlation coefficient); red indicates less similarity. Populations that are more closely related tend to cluster together. Population abbreviations: ACB, African Caribbeans in Barbados; ASW, Americans of African Ancestry in SW USA; BEB, Bengali from Bangladesh; CDX, Chinese Dai in Xishuangbanna, China; CEU, Utah Residents (CEPH) with Northern and Western European Ancestry; CHB, Han Chinese in Beijing, China; CHS, Southern Han Chinese; CLM, Colombians from Medellin, Colombia; ESN, Esan in Nigeria; FIN, Finnish in Finland; GBR, British in England and Scotland; GIH, Gujarati Indian from Houston, Texas; GWD, Gambian in Western Divisions in the Gambia; IBS, Iberian Population in Spain; ITU, Indian Telugu from the UK; JPT, Japanese in Tokyo, Japan; KHV, Kinh in Ho Chi Minh City, Vietnam; LWK, Luhya in Webuye, Kenya; MSL, Mende in Sierra Leone; MXL, Mexican Ancestry from Los Angeles USA; PEL, Peruvians from Lima, Peru; PJL, Punjabi from Lahore, Pakistan; PUR, Puerto Ricans from Puerto Rico; STU, Sri Lankan Tamil from the UK; TSI, Toscani in Italia; YRI, Yoruba in Ibadan, Nigeria

The main goal of the Chromosome 1 runs is to compare the scalability of the parallel algorithms and to assess the efficiency of the OmegaPlus binary-based analysis in terms of both execution time and accuracy on a large, real-world dataset. Consequently, using the full dataset of Chromosome 1, we do not aim to detect selective sweeps or discuss the biological significance of the findings because the entire dataset represents a mixture of several populations, thus violating certain assumptions of OmegaPlus and neutrality tests in general. However, we analyzed each population separately to demonstrate the usage of OmegaPlus on subsets of the entire dataset. Given that this analysis is biologically meaningful and may provide information related to local adaptation, the OmegaPlus output reports are available for download in Additional file 2, and the scores along Chromosome 1 for each population are plotted in Additional file 3. Furthermore, for each pair of populations, we calculated the Spearman correlation coefficient between their OmegaPlus scores to generate the heatmap that is shown in Fig. 12. The heatmap demonstrates that more closely related populations are more similar in terms of patterns of their OmegaPlus scores. This finding probably reflects the more extensive coancestry of more closely related populations (e.g., African populations) compared with more distant populations (e.g., African versus European populations). Alternatively, it might represent common selective pressures between related populations. Further examination is required, which, however, is beyond the scope of this work.

#### Comparison with other neutrality tests

A recent study by Crisci et al. [38] assessed the performance of various implementations, including OmegaPlus, to detect selection for different biologically relevant scenarios such as population size changes. The findings revealed that, in terms of power to reject the neutral hypothesis under both equilibrium and non-equilibrium conditions, OmegaPlus outperforms all alternative implementations considered in the study, i.e., SweepFinder [22], SweeD [26], and iHS [39]. As a complementary comparison, we conduct here a performance evaluation of these tools in terms of analysis time. For this comparison, we use Hudson’s ms [34] to generate simulated datasets with sample size m = (100, 1,000) and number of sites k = (10,000, 100,000). Table 4 presents execution times for sequential and parallel runs with 32 threads.

**Table 4:** Execution times (in seconds) for the analysis of simulated datasets using OmegaPlus, SweeD, SweepFinder, and selscan (iHs)

Sequences	SNPs	Threads	OmegaPlus	SweeD	SweepFinder	Selscan (iHS)
100	10,000	1	5.7	124.7	540.4	858.5
		32	0.7	4.7	–	38.6
	100,000	1	652.9	1,169.0	4,138.1	57,996.9
		32	21.7	36.3	–	2,442.1
1,000	10,000	1	7.1	283.5	132,938.2	37,340.9
		32	1.3	60.6	–	1,428.8
	100,000	1	753.1	1,345.5	135,996.3	433,811.2
		32	28.8	74.7	–	14,834.2

The generic algorithm is used for the parallel execution of OmegaPlus. SweepFinder does not use multiple threads

As can be observed in Table 4, OmegaPlus outperforms all other tools for both the sequential and the parallel runs. Such a comparison is useful for choosing which software to deploy, particularly for large datasets. However, these tools are fundamentally different, in terms of both the methods they implement and/or the type of selective sweeps they detect. For instance, SweeD and SweepFinder rely on the composite likelihood ratio and detect complete selective sweeps (OmegaPlus also detects complete selective sweeps) by analyzing the site frequency spectrum (SFS). However, they require a significantly larger number of floating-point operations than OmegaPlus. Further, the iHS algorithm of the selscan [40] software uses extended haplotypes to detect ongoing (incomplete) selective sweeps.

### Sensitivity and specificity

#### Experimental setup

To assess the sensitivity and accuracy of OmegaPlus, we use Hudson’s ms and mssel tools to generate neutral datasets and datasets with selection, respectively. Both tools perform coalescent simulations with past demographic changes. Given the trajectory of a beneficial mutation, mssel simulates datasets under the simultaneous effect of demography and positive selection. The trajectory of the beneficial mutation is constructed using the same demographic model as the polymorphic dataset that it generates. A total of 1,000 replications was performed for each of the demographic models described in Table 5. The bottleneck models 1–4 were used for the comparison between OmegaPlus and a series of summary statistics. Additional simulations (bottleneck models 1–7) were used to demonstrate the effect of the *-minwin* parameter of OmegaPlus. The command lines for both ms and mssel tools are provided in Additional file 4.

**Table 5:** Parameter values of the simulations

Demographic model	Beginning time of bottleneck	Ending time of bottleneck	Relative population size during bottleneck	Relative population size after bottleneck
constant model	NA	NA	NA	NA
bottleneck1	0.100	0.1004	0.5	1.0
bottleneck2	0.015	0.0160	0.5	1.0
bottleneck3	0.015	0.0170	0.01	1.0
bottleneck4	0.015	0.0160	0.005	1.0
bottleneck5	0.015	0.0170	0.1	1.0
bottleneck6	0.010	0.0104	0.5	1
bottleneck7	0.010	0.0104	0.1	1

Time is measured backwards, from the present to the past. Thus, the ‘beginning’ of the bottleneck is the most recent time point that the population size changes. Forward in time, it corresponds to the ‘end’ of the bottleneck

The total length of each simulated genome is assumed to be 1,000,000 bp. The length value is arbitrary, because ms and mssel adopt the infinite site model; it is used only to allow the illustration of the results in a similar way to real datasets (whose size is finite). The genome was split in 1,000 overlapping windows of length 10,000 bp each. The distance between the starting points of two consecutive windows (offset) is 1,000 bp. Several summary statistics were evaluated at each window, for both the neutral and the selection dataset. To set the significance threshold for each summary statistic, we performed a parametric bootstrapping procedure that is often used in selection scans [22]: for a given demographic model (e.g. bottleneck1), we calculated the minimum value of each summary statistic among the sliding windows. We conducted 1,000 replications for each demographic model, obtaining 1,000 minimum values for each summary statistic. The significance threshold of each summary statistic was set as the 5th percentile of its minimum values. Given that we consider only the minimum values for the calculation of the threshold value (i.e., the minimum value per replication), there is no multiple testing problem that could result from the number of windows.

#### Comparison with summary statistics

Nowadays, several methods (neutrality tests) exist for detecting selective sweeps. The majority of such tests are simple summary statistics whose value is different between genomic regions that have experienced a selective sweep and regions that are located far away from a selective sweep. Some of them, for example, Tajima’s D [41], Fu & Li D ^∗^ [42], and H ^′^ [43], use information from the site frequency spectrum. A second class of tools, such as Hudson’s C [44] and Depaulis and Veuille K & H [45], use information that is mostly related to linkage disequilibrium or the polymorphism level. Finally, there are statistics, for example, *θ*_*π*_ [46], whose value is based only on the polymorphism level. We compared OmegaPlus with the following statistics: (i) *θ*_*π*_, (ii) H ^′^, (iii) Tajima’s D, (iv) Fu & Li D ^∗^, (v) Fu & Li F ^∗^ [42], (vi) Depaulis and Veuille (1998) H, and (vii) Hudson’s C statistic. *θ*_*π*_ measures the level of nucleotide diversity by calculating the average number of pairwise differences between sequences, thus it is expected to be low in windows nearby a selective sweep. The H^′^ statistic is defined as H^′^=(*θ*_*π*_−*θ*_*H*_)/*Var*(*θ*_*π*_−*θ*_*H*_), where *θ*_*H*_ [47] is an estimator of the *θ*=4*Nμ* parameter (*μ* is the mutation rate per site per individual) based on the site homozygosity. H ^′^ assumes low values in the proximity of a selective sweep. Tajima’s D measures a normalized difference between *θ*_*π*_ and Watterson’s *θ*_*W*_ [48] and assumes low (negative) values in the proximity of a selective sweep. The same is true for Fu & Li D ^∗^ and Fu & Li F ^∗^. Depaulis and Veuille H measures the haplotype heterozygosity, thus it also decreases nearby a selective sweep. Finally, Hudson’s C is an estimate of the population recombination rate *rho*=4*Nc*, where *c* is the recombination rate per bp per individual. The estimate is based on the variance of the site frequencies. Thus, its value mostly depends on the level of LD and assumes low values in the proximity of a selective sweep.

**Fig. 13:**
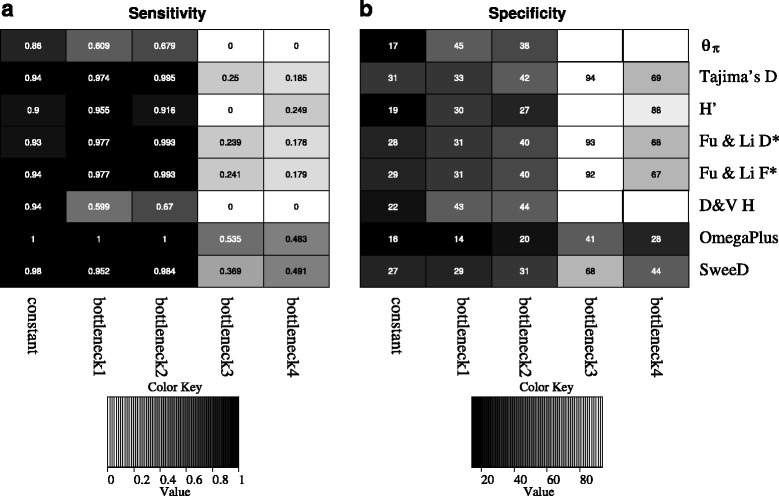
Comparison between OmegaPlus and other commonly used summary statistics and neutrality tests. **a** Sensitivity analysis, i.e., the percentage of the simulations with selection that are detected as selected by each tool. **b** Specificity analysis, i.e., the distance of the detected target of selection from the true target of selection (in kilobases). The darker the cell in the heatmap is, the greater the accuracy is

We compared the sensitivity and the specificity of these tools to detect selective sweeps for various demographic models. As sensitivity, we define the percentage of the replications of each model with selection in which the value of the neutrality test was more extreme than the threshold value. As specificity, we calculate the distance between the reported selective sweep region and the true target of selection. We tested the performance of the neutrality tests on several demographic models (model parameters are provided in Table 5; the command lines can be found in Additional file 4). The first model is a constant population size model. For all summary statistics, even the very simple ones, such as *θ*_*π*_, both sensitivity and specificity are high (Fig. 13). Sensitivity and specificity are similarly high for mild bottlenecks as well. For example, for bottleneck2, in which the population size during bottleneck becomes half of the present population size, *θ*_*π*_ and Depaulis and Veuille H can identify approximately 67 % of the sweeps. On average, the sweep locations reported by these tools were 38 kb and 44 kb away from the true target of selection, respectively. All other neutrality tests performed better regarding sensitivity. OmegaPlus can identify all selective sweeps and its average reported distance from the true target of selection is 20 kb (closer than any other neutrality test). For more severe bottlenecks, the performance of all tools is considerably reduced. For instance, for bottleneck4 (relative population size during bottleneck is 0.005 of the present-day population), OmegaPlus can detect only slightly less than half (48.3 %) of the sweeps, at an average distance of 28 kb from the true target of selection. Other neutrality tests performed even worse. For example, Tajima’s D detected only 18.5 % of the sweeps, at a distance of approximately 70 kb away from the real target of selection.

A study by Pavlidis et al. [27] showed that combining LD- and SFS-based methods can potentially increase our ability to detect selective sweeps and reduce the false positive rate. The rationale behind their approach is that both LD-based tests (such as OmegaPlus) and SFS-based tests (such as SweeD) should point to the same location when a selective sweep has occurred. To facilitate the combination of results, we have implemented two R scripts that illustrate both OmegaPlus and SweeD results in a common plot. Both of the scripts highlight outliers detected by both OmegaPlus and SweeD. The download links to the R scripts are provided in the Additional file 4.

The first R script invokes OmegaPlus and SweeD to analyze a dataset using the same grid size *g* for both tools. The *g* points are defined as *g*_*i*_=(*o*_*i*_,*s*_*i*_), where *o*_*i*_ and *s*_*i*_ are the OmegaPlus and SweeD values of the grid-point *i*. Given a significance level *a*, e.g., 5 %, the threshold value to detect outliers is defined as the point *g*_*c*_=(*o*_*c*_,*s*_*c*_) for which 5 % of grid-points exist where both OmegaPlus and SweeD values are greater than *o*_*c*_ and *s*_*c*_, respectively. The second R script calculates the threshold via simulations instead of relying on the input dataset.

#### Analysis of input parameters

As already mentioned, to perform an OmegaPlus analysis, a minimum number of five input arguments are required. There are also optional parameters that can be used, such as the minimum number of SNPs required in a region needed calculate the *ω*-statistic (*-minsnps*), the maximum difference (balance parameter) in terms of number of SNPs between the left and right windows (*-b*), or whether singletons should be considered for the computations (*-no-singletons*).

Some input parameters have minimal effect on the outcome while others affect the results profoundly. The *-maxwin* parameter, for instance, which specifies the maximum extent of a sweep, has negligible effect on the results if it is sufficiently large. For example, for mutation and recombination rates similar to *Drosophila melanogaster*, all *-maxwin* values greater than 50 kb will give approximately the same results. This is because it is almost certain that the *ω*-statistic maximizes for a window smaller than the *-maxwin* value. The balance parameter *-b* restricts the left and right windows to be approximately equal in size. Thus, calculations on significantly uneven windows in terms of number of SNPs are avoided. Similarly to *-maxwin*, the value of *-b* does not have a great effect on the results. However, computations become faster when a low *-b* value is provided, e.g., less than 10.

The *-no-singletons* flag is necessary only when we suspect that the majority of singletons result from sequencing errors. It is not advised to exclude singletons from high quality datasets because the power to detect selective sweeps will decrease. Singletons in abundance is essentially the main signature of a selective sweep, and thus excluding them from the analysis will reduce our ability to detect a sweep.

**Fig. 14:**
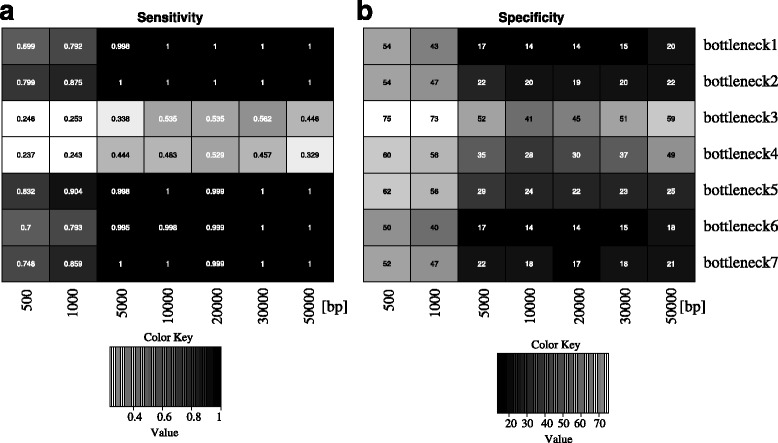
Sensitivity and specificity analysis for various *minwin* values and bottleneck scenarios. **a** Heatmap for the percentage of simulations with selection detected as selected by OmegaPlus for various *minwin* sizes and bottleneck scenarios. **b** Distance of the detected target of selection from the true target of selection. In **a**, darker color means higher sensitivity, i.e. more cases of selection are detected. In **b**, darker color means higher accuracy, i.e. the detected target of selection is closer to the real target of selection

The minimum number of SNPs (*-minsnps*) and the minimum window size (*-minwin*) parameters can affect the results dramatically. Small values, such as 2 for *minsnps*, and 500 bp for *minwin* (when human data are analyzed), must be avoided. This is because such values for these parameters increase the stochasticity of the results significantly. Even under neutrality, it is highly possible that linkage disequilibrium will assume very high values in small windows. The *minsnps* parameter has a similar effect. It is a challenge to provide a guideline for setting these parameters because their values should be dataset-specific. Further, they depend on factors such as recombination and mutation rate and on the demographic history of the organism. When the evolutionary parameter values are such that they increase the variance along the genome (e.g., small recombination rate, small population size, bottleneck, or small migration rate), high *minwin* and *minsnps* values are preferable. Typically, we set *minsnps* to 5 and *minwin* to 10 kb for human data. The corresponding values for *Drosophila* are usually smaller. Figure 14 demonstrates the effect of *minwin* on the sensitivity and specificity of OmegaPlus. As can be observed in the figure, small window sizes (e.g., 500 bp) reduce performance. The reason behind this observation is that stochasticity increases in small windows, outperforming the effect of a selective sweep. On the other hand, large *minwin* values (e.g., 50 kb or larger) would also reduce the performance of the algorithm because they contain a large proportion of SNPs not affected by the selective sweep. Determining the optimal *minwin* value is not feasible, because the choice is affected by the demographic model. For example, all *minwin* values between 10 kb and 50 kb perform excellently for bottleneck1, whereas a *minwin* value of 30 kb gives the best results for bottleneck3.

### Conclusions

With the introduction of the generic parallelization alternative in the OmegaPlus source code, there are currently four algorithmic variations that one can use, raising the question: “Which parallelization alternative is the most suitable for my datasets?”. A prerequisite to answering this question is understanding which factors affect performance and which factor is better served by each parallelization alternative. The three dataset-dependent factors that have an impact on performance are the sample size, the total amount of polymorphism, and the distribution of SNPs along a dataset. The performance of the fine-grained approach (OmegaPlus-F) improves with an increasing sample size. This is because OmegaPlus-F exploits parallelism in narrow genomic regions (maximum region that a selective sweep can affect), where the number of SNPs is limited and the only factor that affects performance is the sample size. In this case, as the sample size increases the computation-to-synchronization ratio improves, yielding better overall performance for OmegaPlus-F. For datasets with increased number of SNPs, the multi-grained approach in OmegaPlus-M is expected to deliver higher performance as the number of SNPs increases because OmegaPlus-M distributes large genomic regions to different threads and each thread analyzes a region uninterrupted, thus eliminating any synchronization overhead. The coarse-grained approach in OmegaPlus-C typically shows similar behavior to OmegaPlus-M. However, OmegaPlus-M is less prone to the distribution of SNPs along the dataset, and as such it should be preferred over OmegaPlus-C at all times. Finally, the performance of the new generic approach, because of the fundamentally different load organization and distribution scheme, remains unaffected by the distribution of SNPs along a dataset. Furthermore, it exploits a region-based processing scheme, similar to the multi-grained approach, which achieves improved performance for large number of SNPs, and implements blocking to improve the computation-to-synchronization ratios observed in OmegaPlus-F for small sample sizes. Overall, the generic approach is the preferred approach for small and moderate size datasets, whereas the n becomes more powerful for large sample sizes, and the multi-grained approach for long genomes.

The discussion so far has not taken into consideration the number of cores. Assuming that the dataset size remains constant as the number of cores increases, each thread is assigned smaller computational load, thus reducing the computation-to-synchronization ratio. Although this is generally the case for all parallel algorithms, the performance degradation becomes less prevalent with an increasing dataset size. The generic approach shows the best performance for increasing number of cores even when the dataset size is not large enough to justify a large number of cores; it has the most stable parallel efficiency over all experiments conducted in this study.

From a performance standpoint, devising an efficient parallel algorithm that scales well for a large number of cores is challenging because of the variety of attributes that the input dataset may have, such as the sample size, the number of SNPs, the data type (binary data or DNA), and the SNP density and distribution in the dataset. All parallel algorithms presented in this study yield qualitatively the same results. However, their performance differs, depending on the aforementioned attributes of the input data. Thus, choosing the right parallel algorithm for an analysis can lead to significantly shorter analysis time and energy savings.

As future work, we intend to further optimize the performance of the kernels used for selective sweep detection by using SIMD (Single Instruction Multiple Data) vector intrinsics such as SSE (streaming SIMD extensions) and AVX (advanced vector extensions), which are available in all modern microprocessor architectures. Computing LD between two SNPs is an embarrassingly parallel problem^2^. Therefore, boosting single-thread execution with vector intrinsics and combining highly optimized kernels with scalable parallel algorithms will lead to significant time reduction for the analysis of large-scale datasets.

Furthermore, we will be exploring emerging hardware architectures such as FPGAs and GPUs for offloading computationally intensive kernels and accelerating LD-based selective sweep detection. As already mentioned, the offload-compute paradigm in the generic algorithm is very suitable for hardware-based acceleration. By appropriately limiting the algorithm’s memory overhead (which is already an input parameter), adapting the memory layout to achieve coalesced memory accesses on a SIMT (single instruction multiple threads) architecture, and devising a simplified OpenCL kernel for LD calculation, we can make use of the computational power of modern GPUs.

Detailed profiling of the current *ω* statistic implementation in OmegaPlus revealed that significant fraction of the total execution time (up to 70 %) is spent calculating LD values between SNPs. Therefore, we intend to devise a reconfigurable accelerator architecture for computing LD, and use high-performance communication infrastructure for hardware acceleration [49, 50] to achieve faster execution when large-scale datasets are analyzed.

Finally, in addition to the way in which the data-reuse optimization is currently deployed for LD and *ω* statistic calculations, the strategy of data reuse can be exploited for the computation of other neutrality tests and summary statistics as well. Although such statistics are not highly compute-intensive, they typically conduct a large number of redundant calculations because of extensive overlaps between neighboring windows. Similarly, the generic processing scheme applied here in the context of selective sweep detection can be adapted to operate on disjoint pairs of genomic regions rather than a single genomic region (as required for selective sweep detection). This will enable the efficient evaluation of long-range LD on whole genomes without conducting redundant computations or requiring excessive memory resources.

## Availability and requirements

**Project name:** OmegaPlus, Software for detecting selective sweeps**Project home page:**https://github.com/alachins/omegaplushttp://pop-gen.eu/wordpress/software**Operating systems:** Linux **Programming language:** C**Other requirements:** none**License:** GNU GPL **Any restrictions to use by non-academics:** none

## Availability of supporting data

Supporting materials can be downloaded from the *GigaScience* GigaDB database [51].

## Endnotes

^1^ A small number of lines of code that are repeated many times during program execution.

^2^ Embarassingly parallel is a term used in parallel computing to describe computational problems that require little effort to distribute the problem to multiple tasks.

## Additional files

Additional file 1 **Human Chromosome 1 OmegaPlus results.** This file contains the OmegaPlus reports and information files for the analysis of the first chromosome of the human genome (2,504 genomes) for two different data representations: DNA data (4 bits per allele) and binary data (1 bit per allele). (ZIP 155 kb)

Additional file 2 **Human Chromosome 1 OmegaPlus results per population.** This file contains the OmegaPlus reports and information files for the analysis of the first chromosome of the human genome per population (26 populations) for DNA data representation. (ZIP 1120 kb)

Additional file 3 **Plots of OmegaPlus scores per population along Human Chromosome 1.** This file contains a series of plots (26 plots, one per population) of the OmegaPlus scores along the human chromosome 1. (ZIP 512 kb)

Additional file 4 **Command lines for ms and mssel, and download links to the R scripts.** This file contains a document that provides the command lines for generating the trajectories of the beneficial mutations in different bottleneck scenarios, the command lines to invoke the software tools ms and mssel for the simulations, and the download links for the R scripts to combine and plot OmegaPlus and SweeD scores. (ZIP 56 kb)
