# Supplementary figures and images for: Scalable linkage-disequilibrium-based selective sweep detection: a performance guide
Source: Gigascience. 2016 Feb 8;5:7. doi: 10.1186/s13742-016-0114-9 (PMC4746822; doi:10.1186/s13742-016-0114-9)

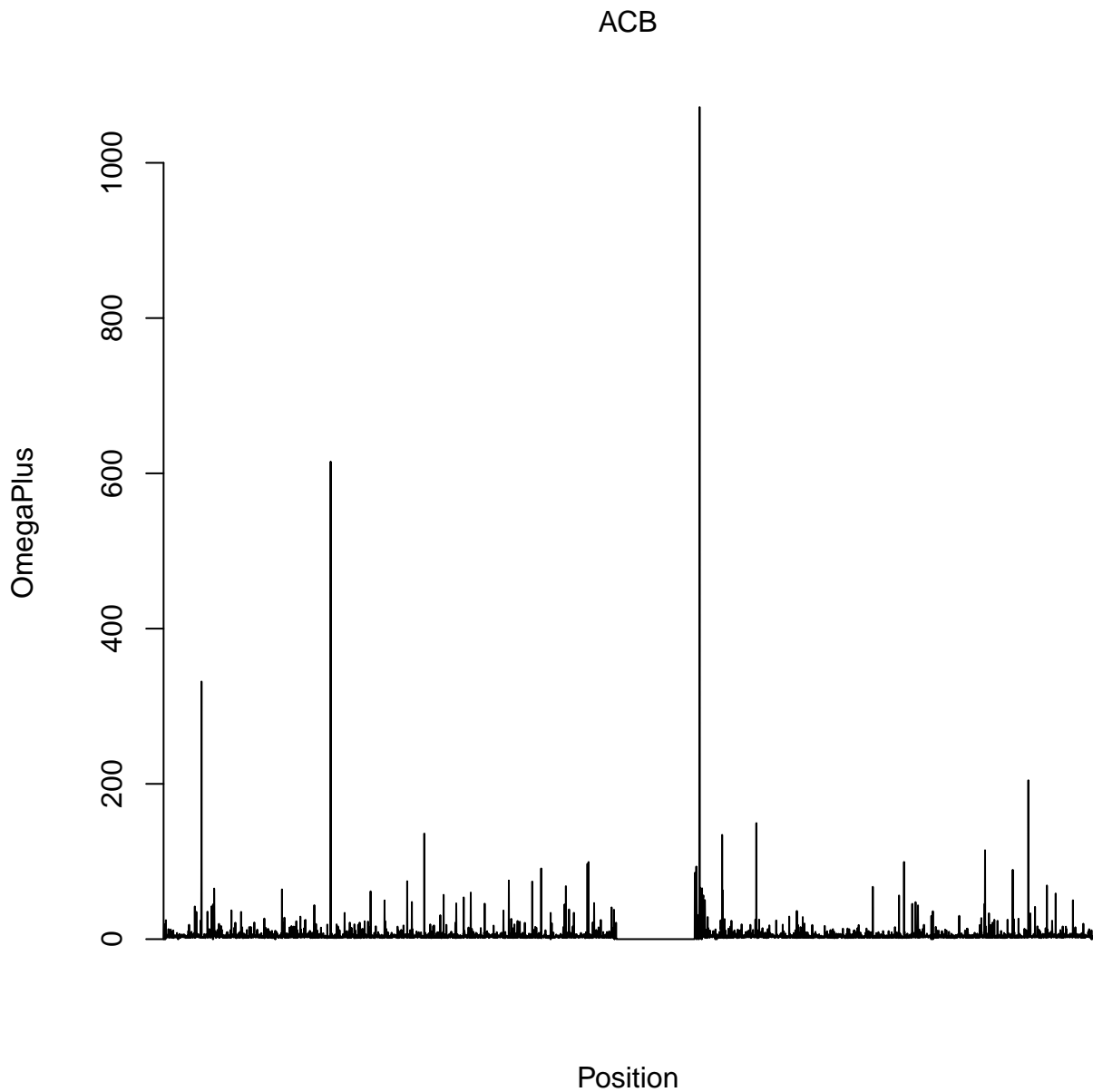

ASW

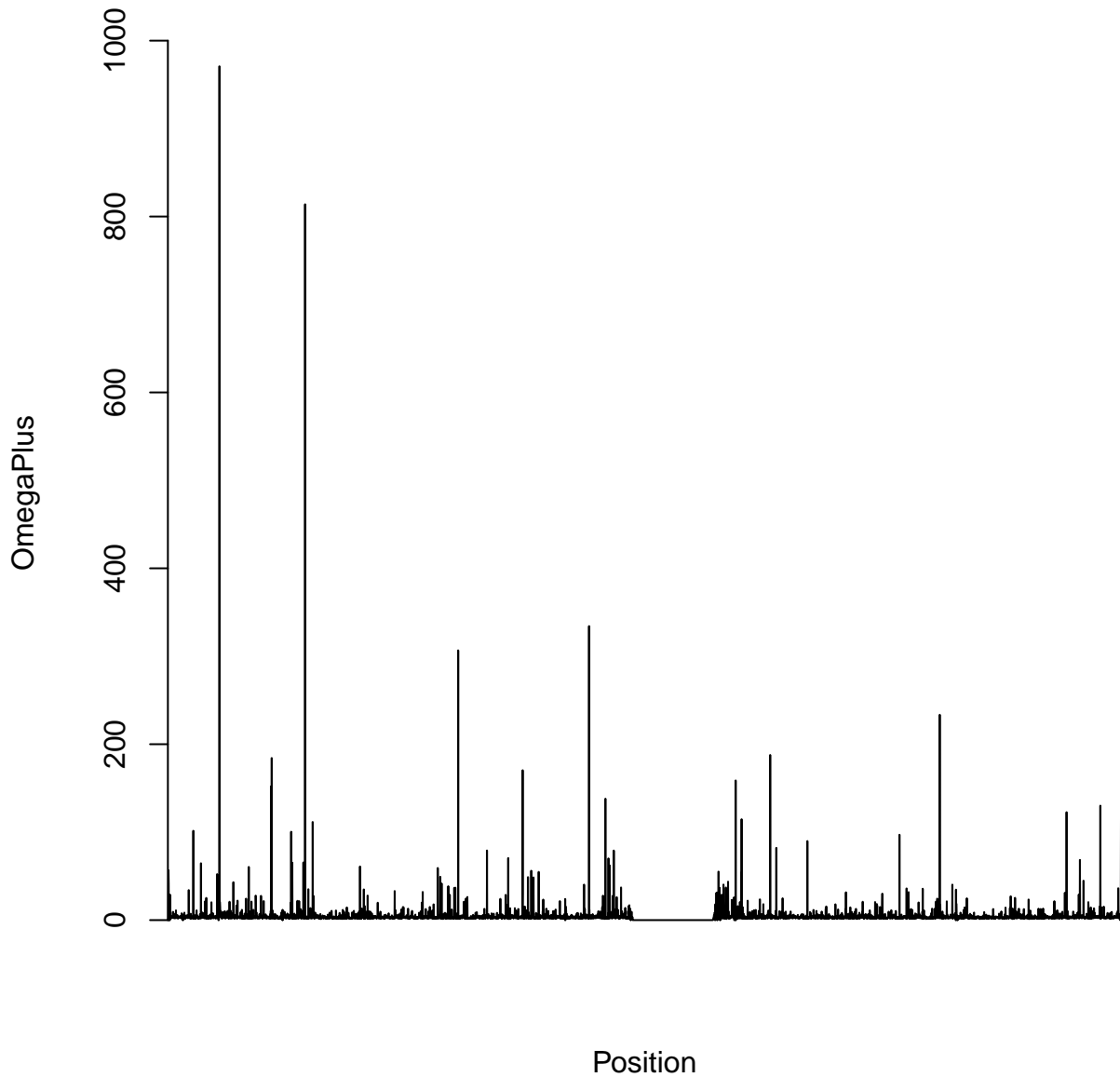

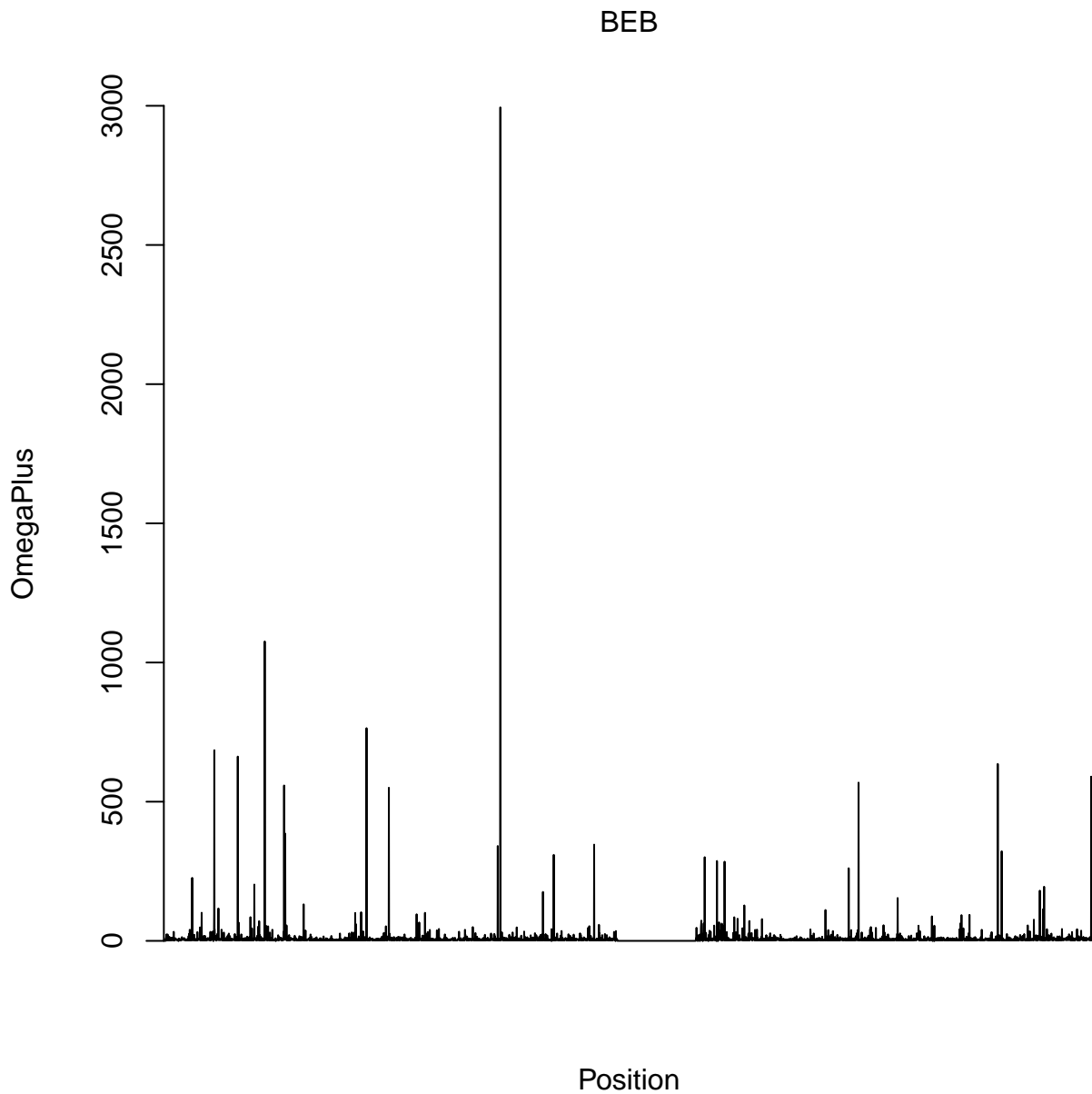

CDX

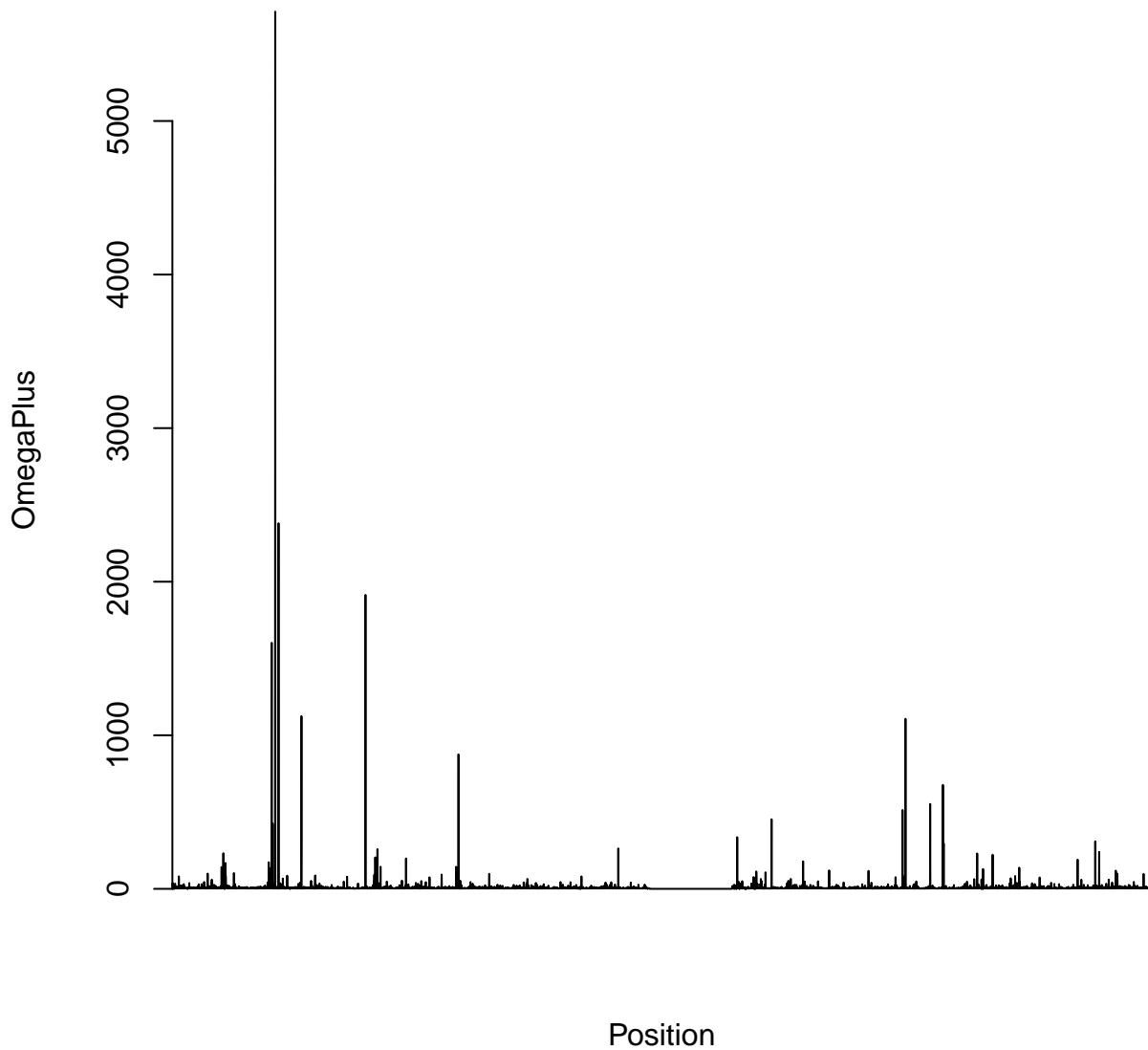

CEU

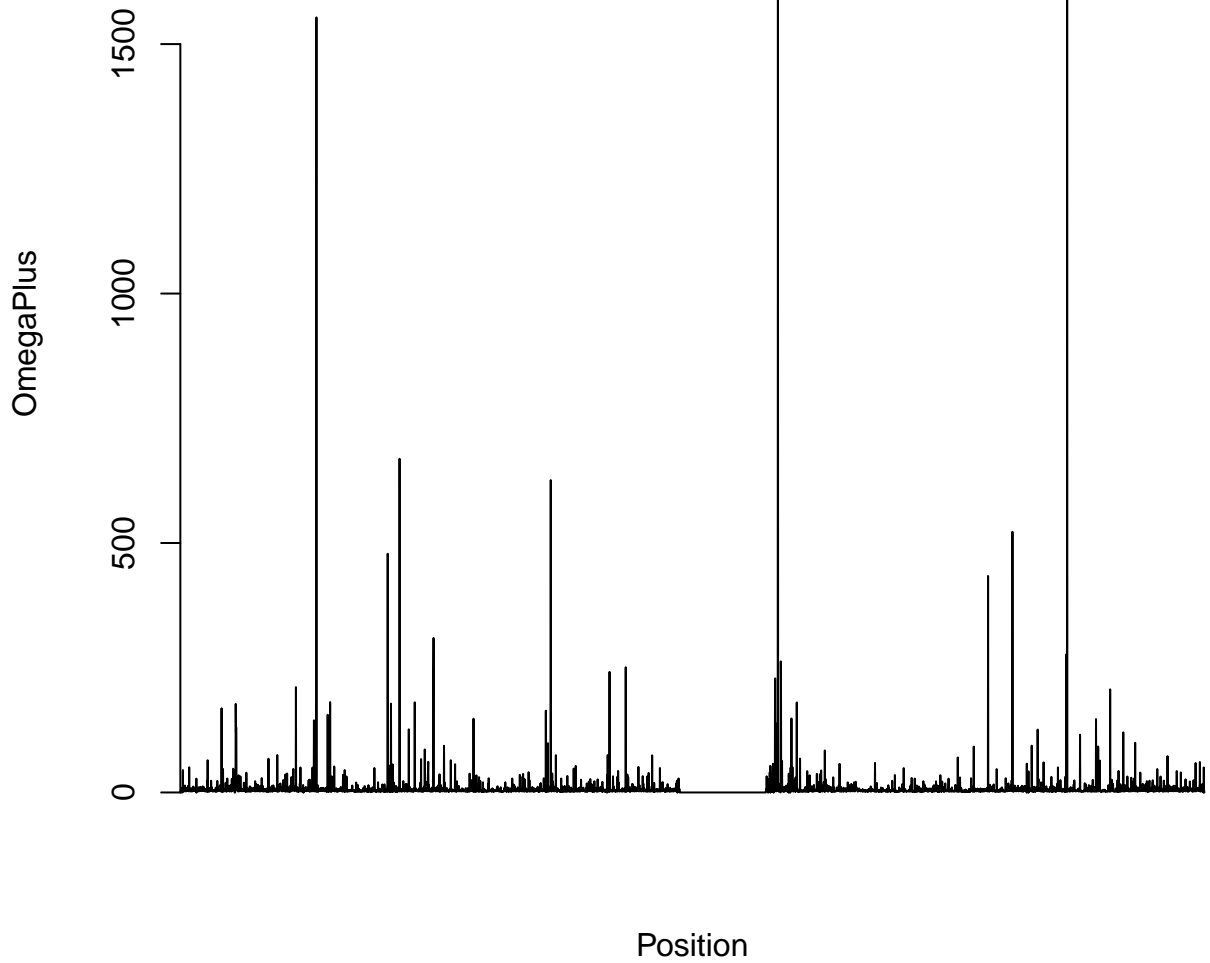

CHB

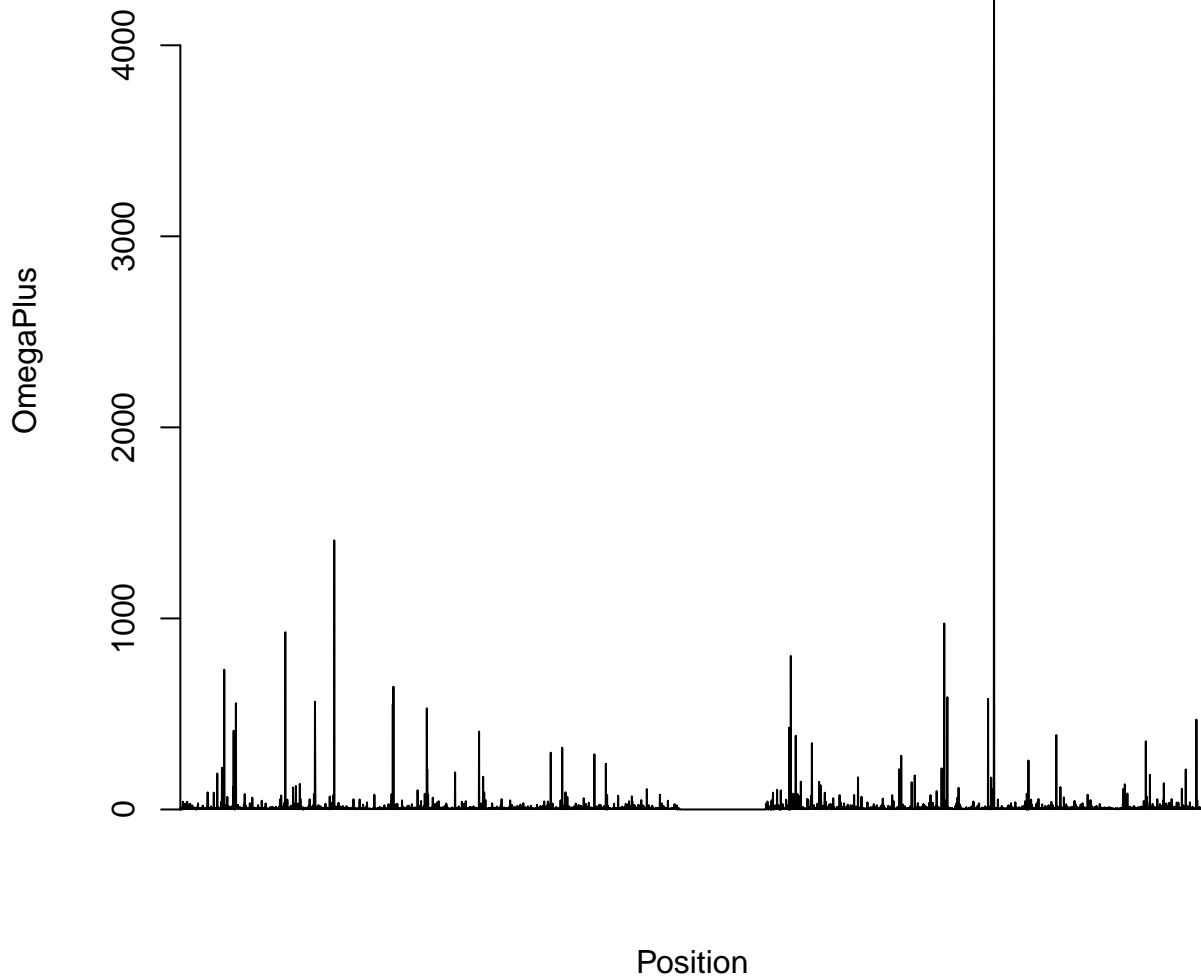

CHS

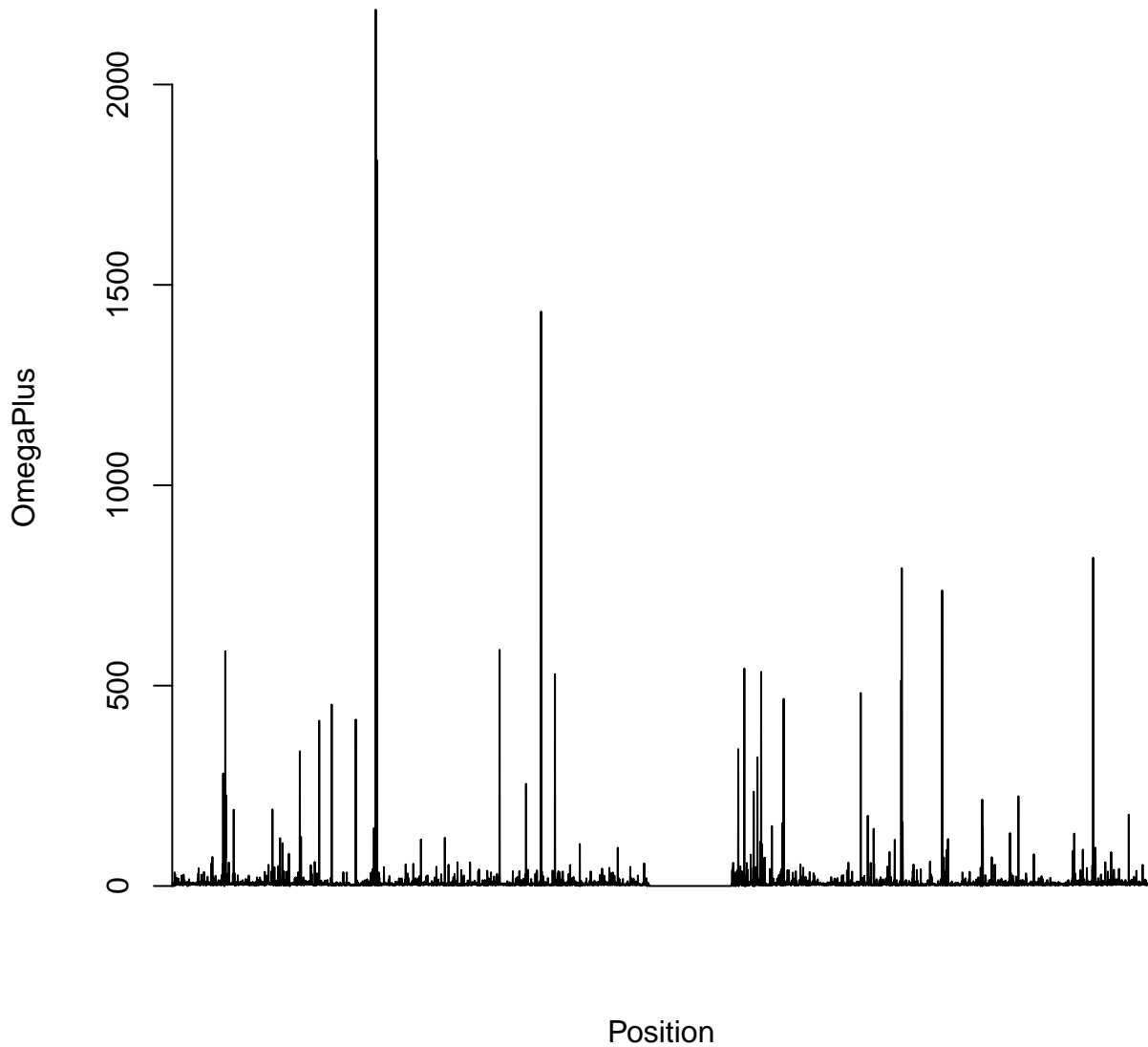

CLM

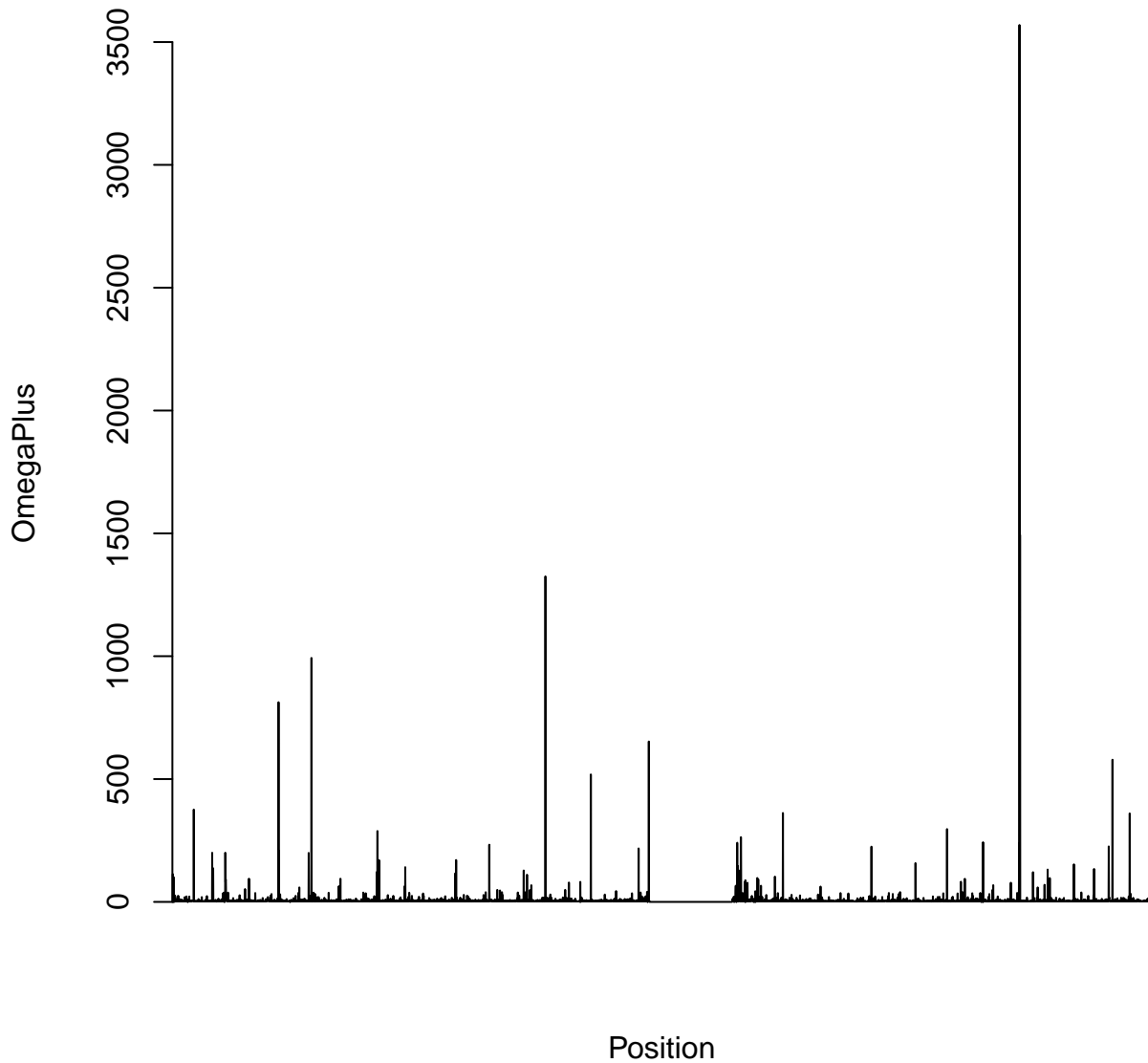

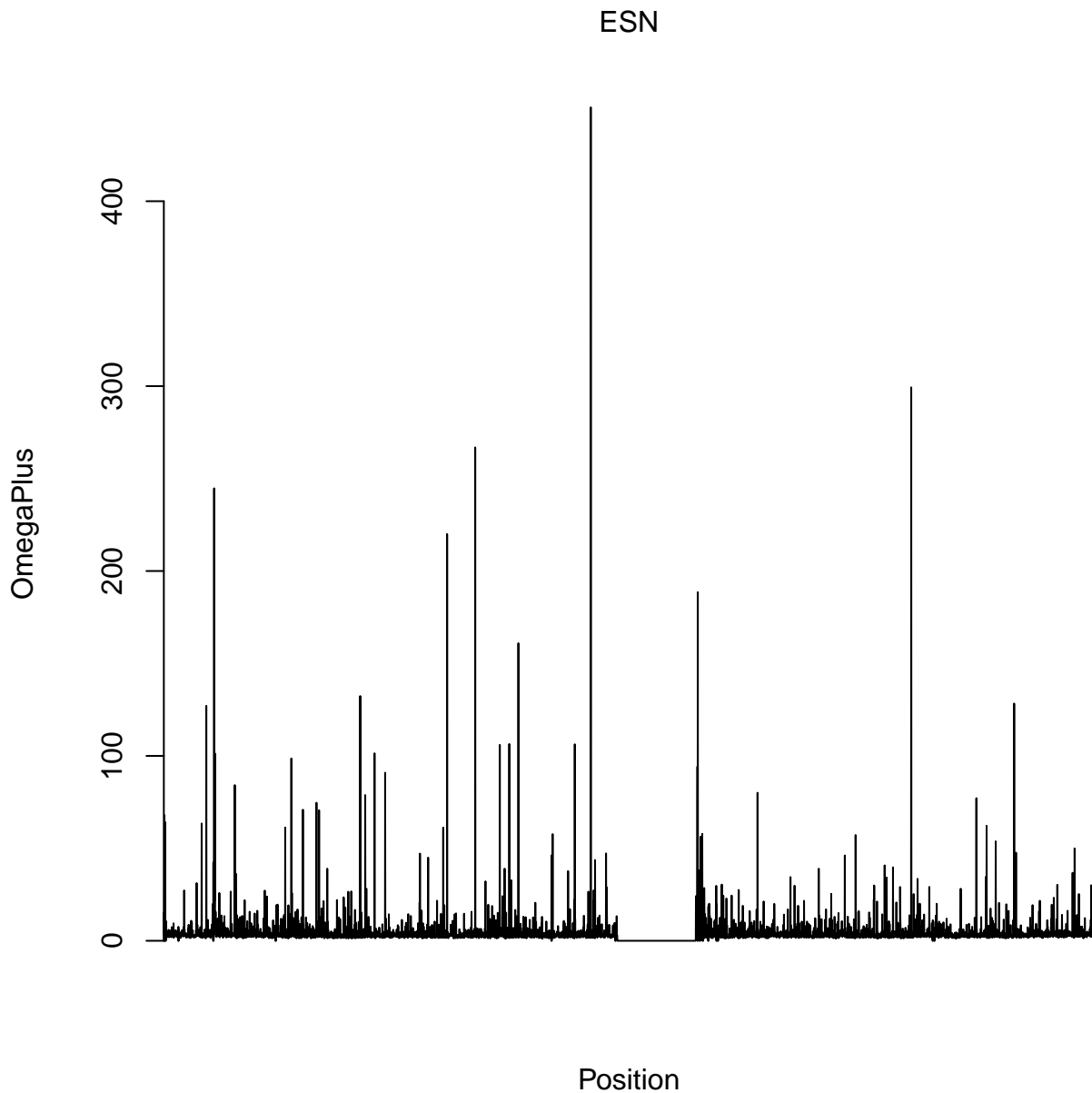

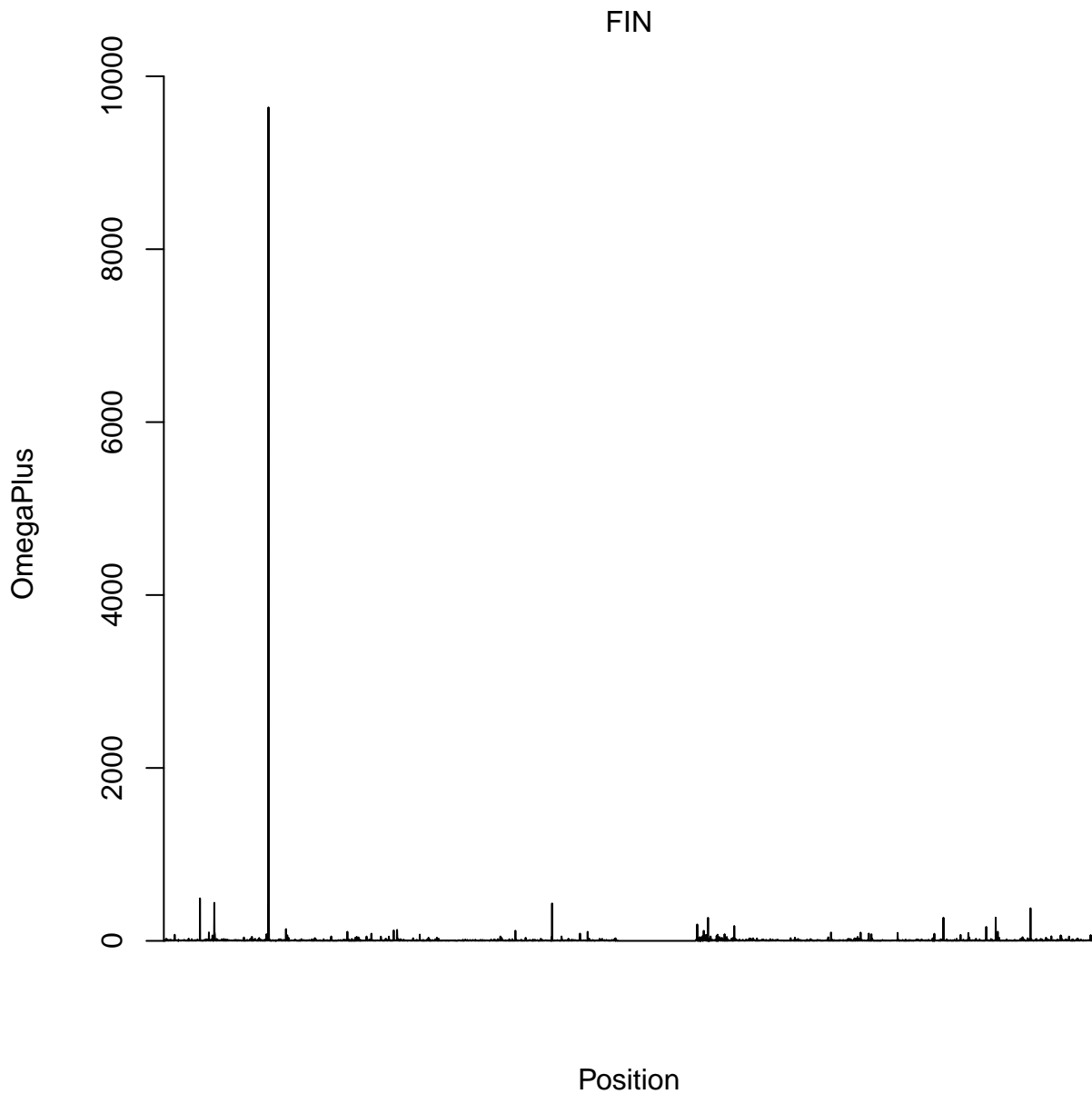

GBR

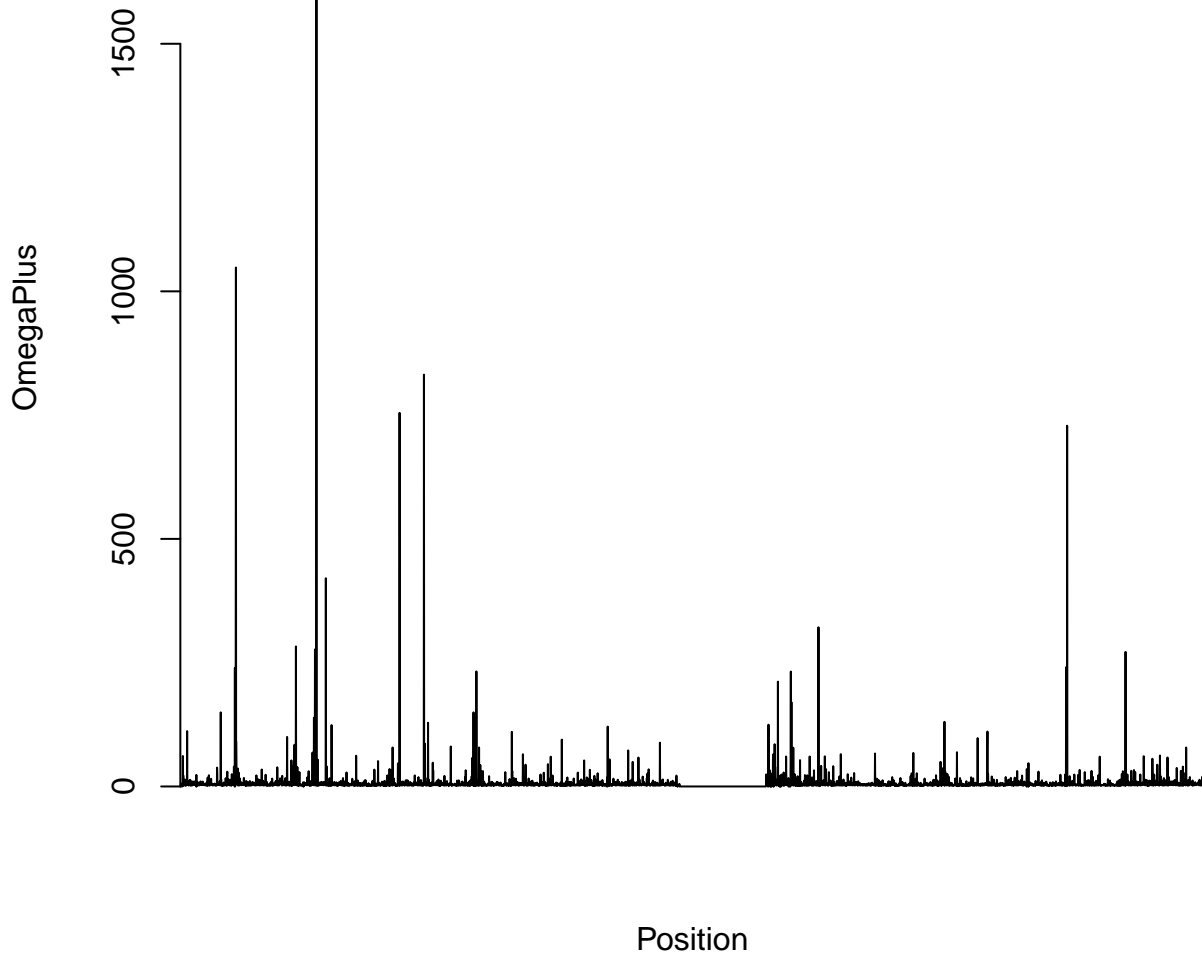

GIH

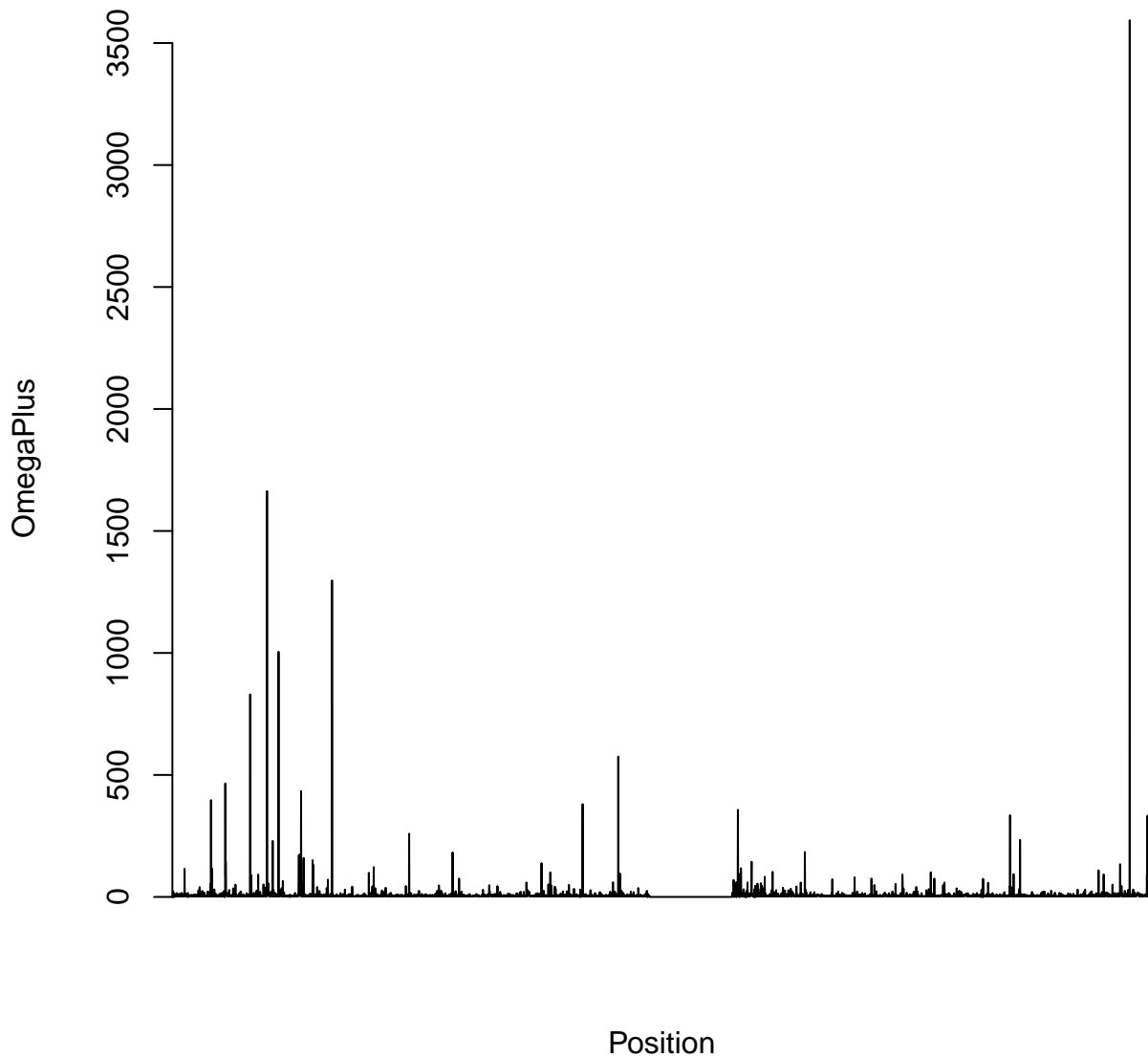

GWD

OmegaPlus

1400  
1200  
1000  
800  
600  
400  
200  
0

Position

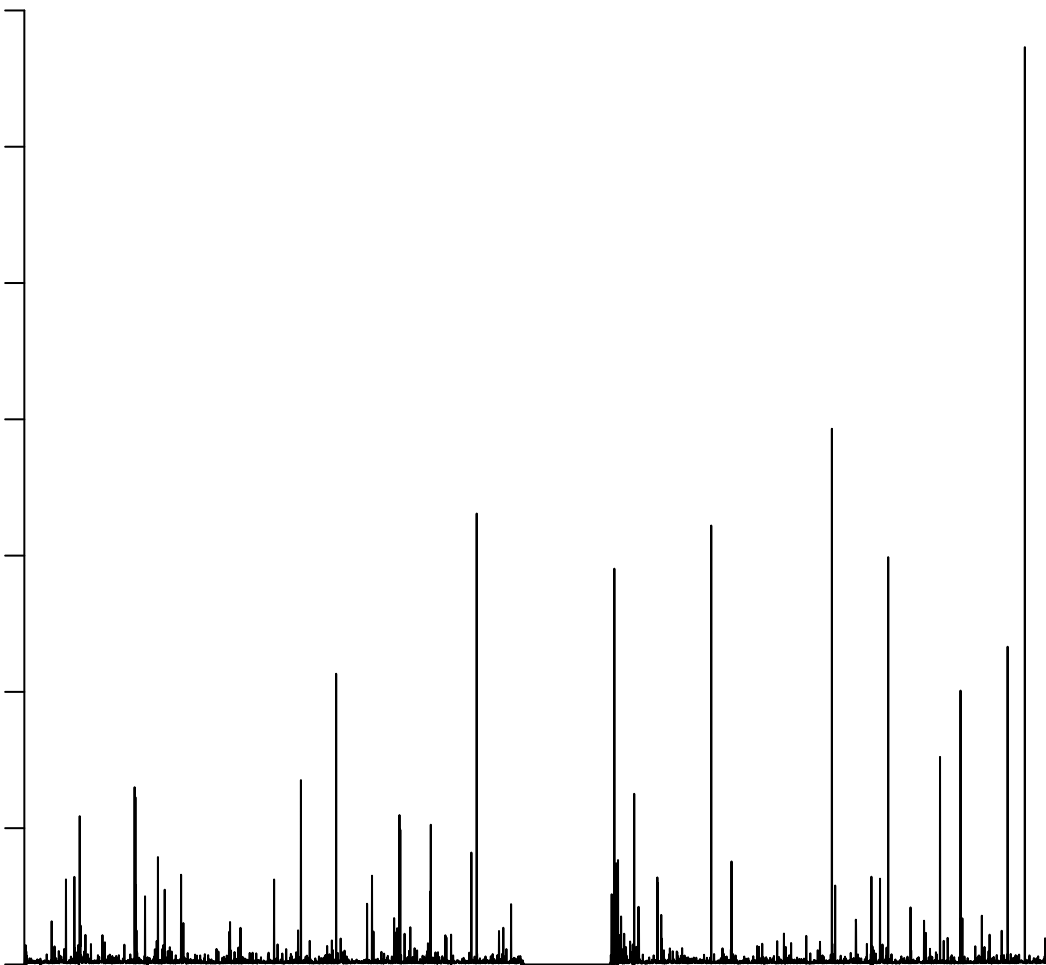

IBS

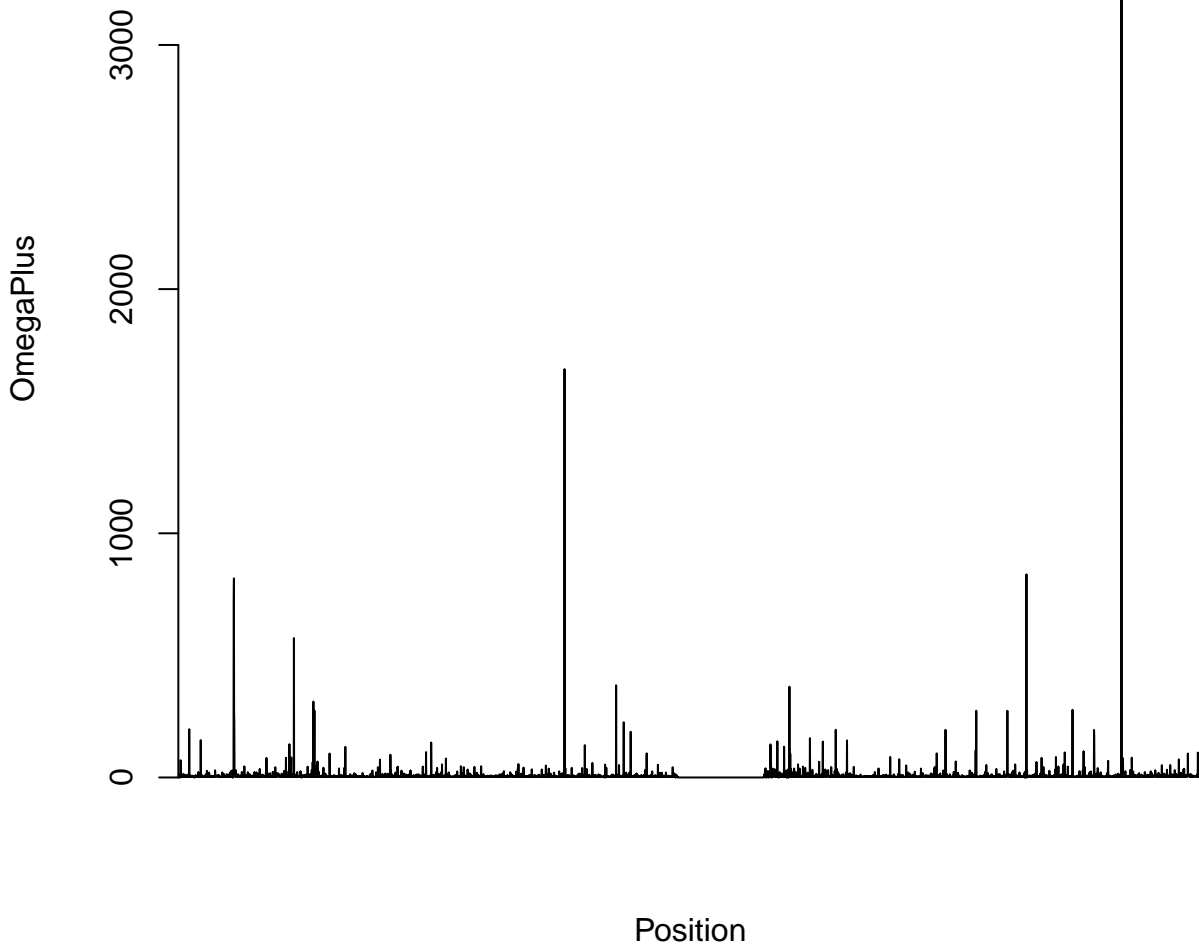

ITU

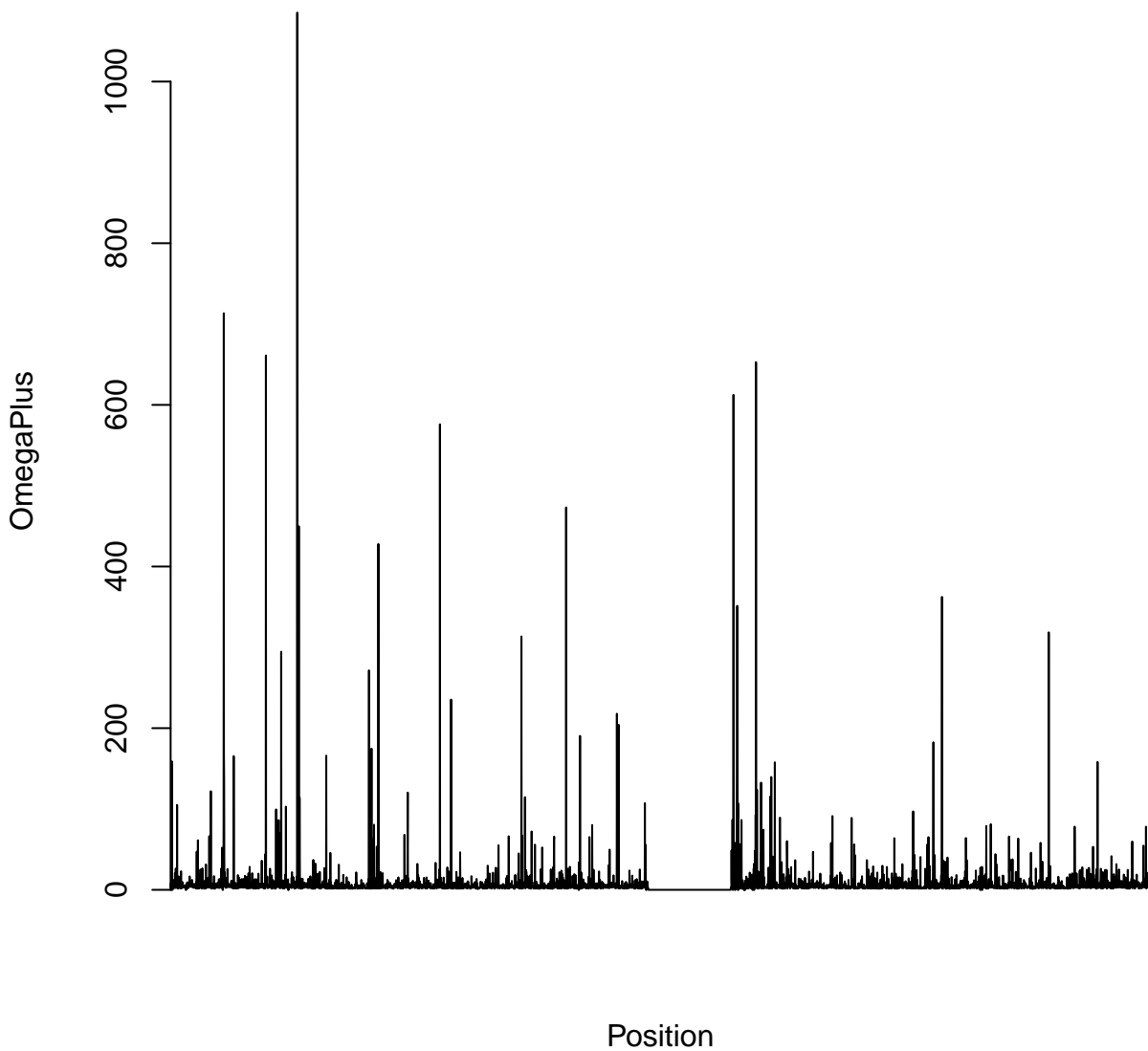

JPT

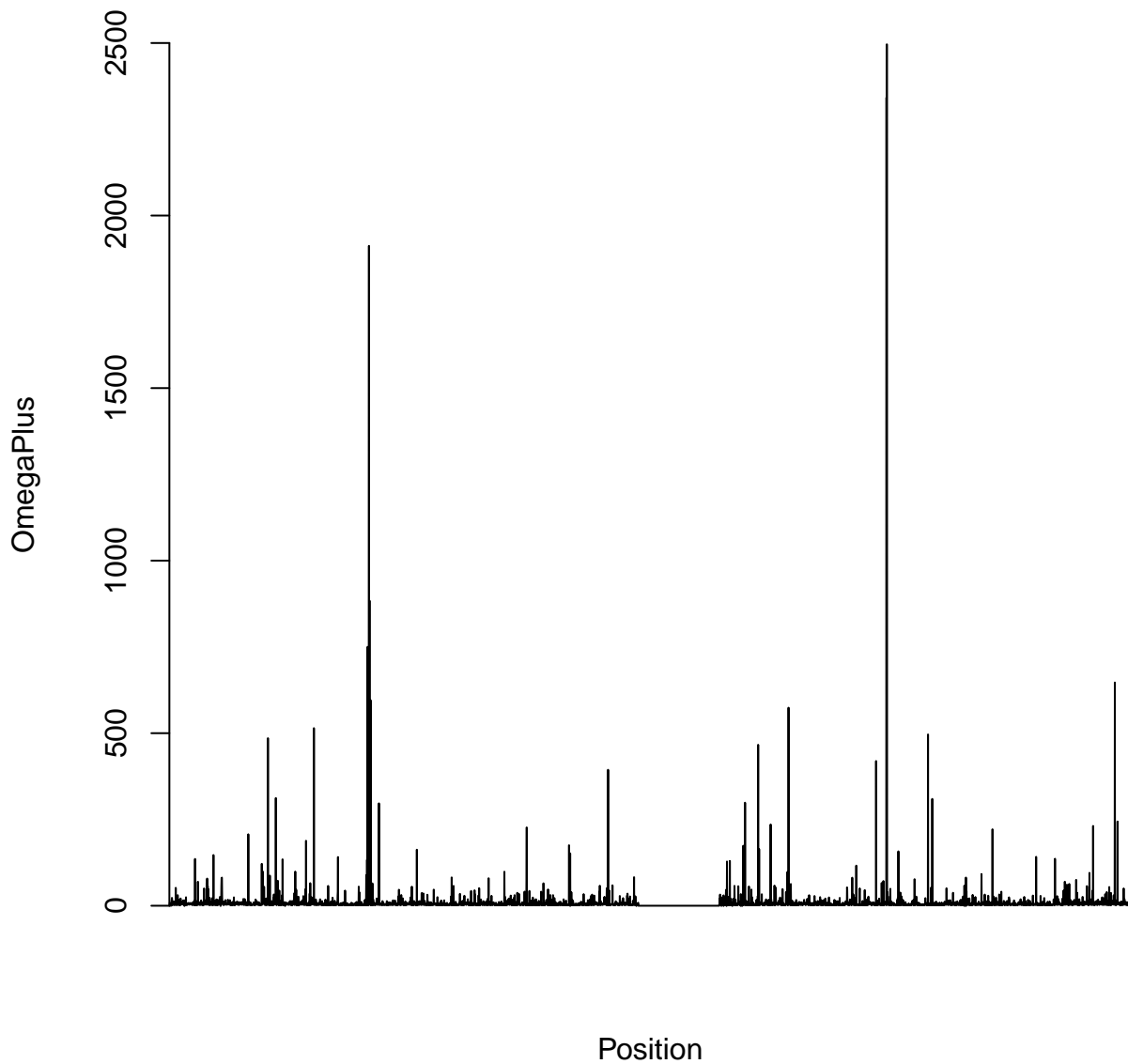

KHV

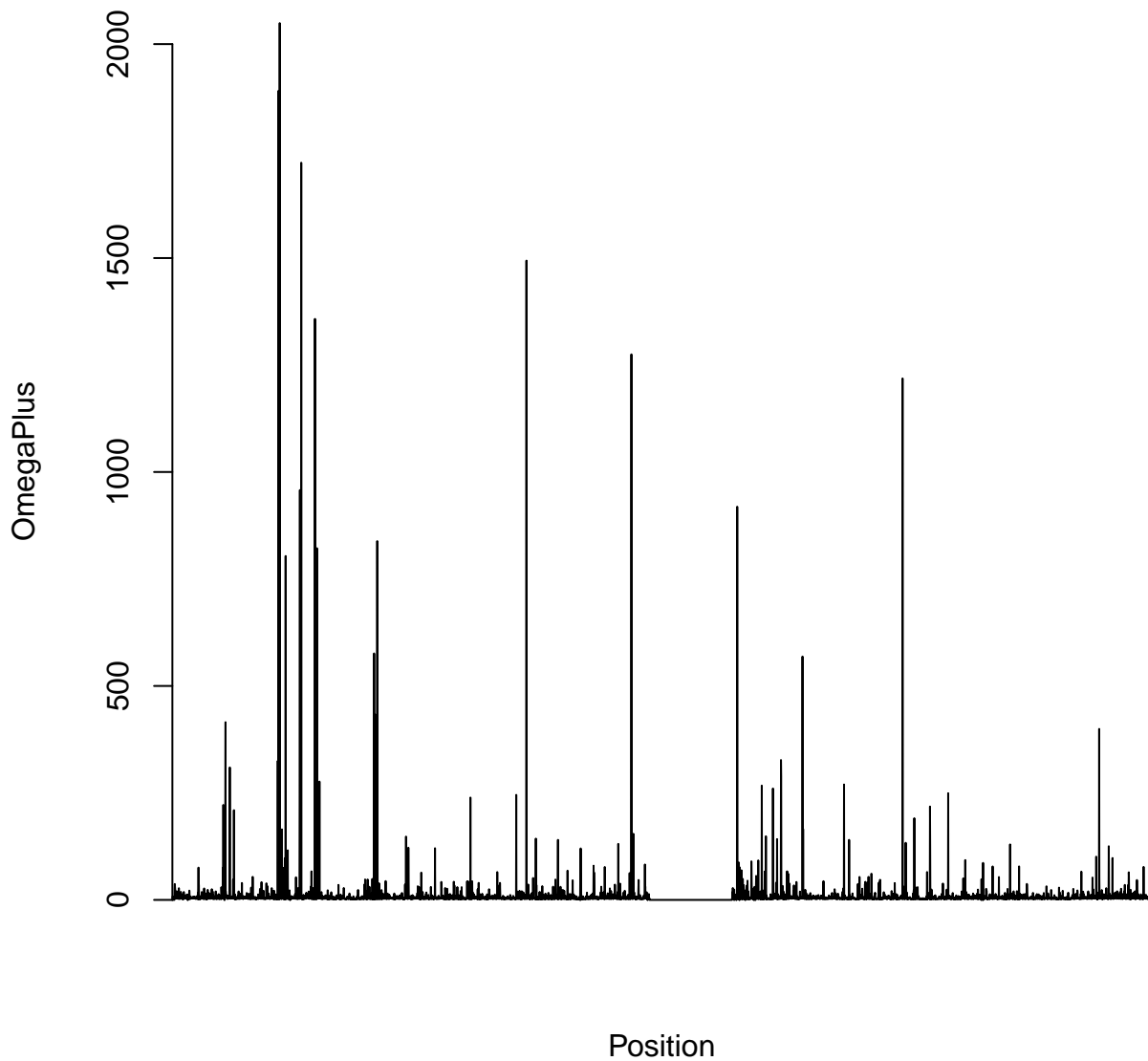

LWK

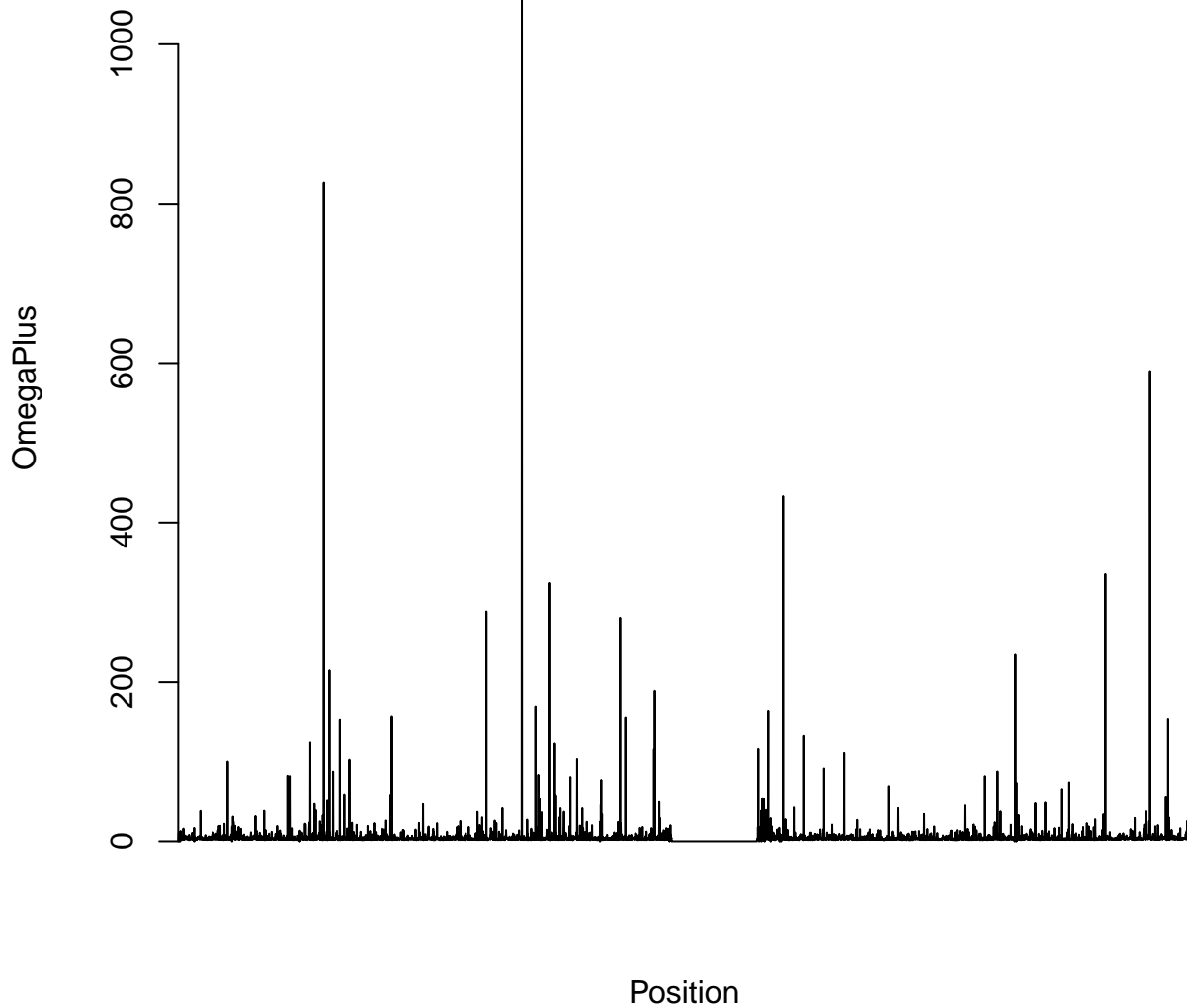

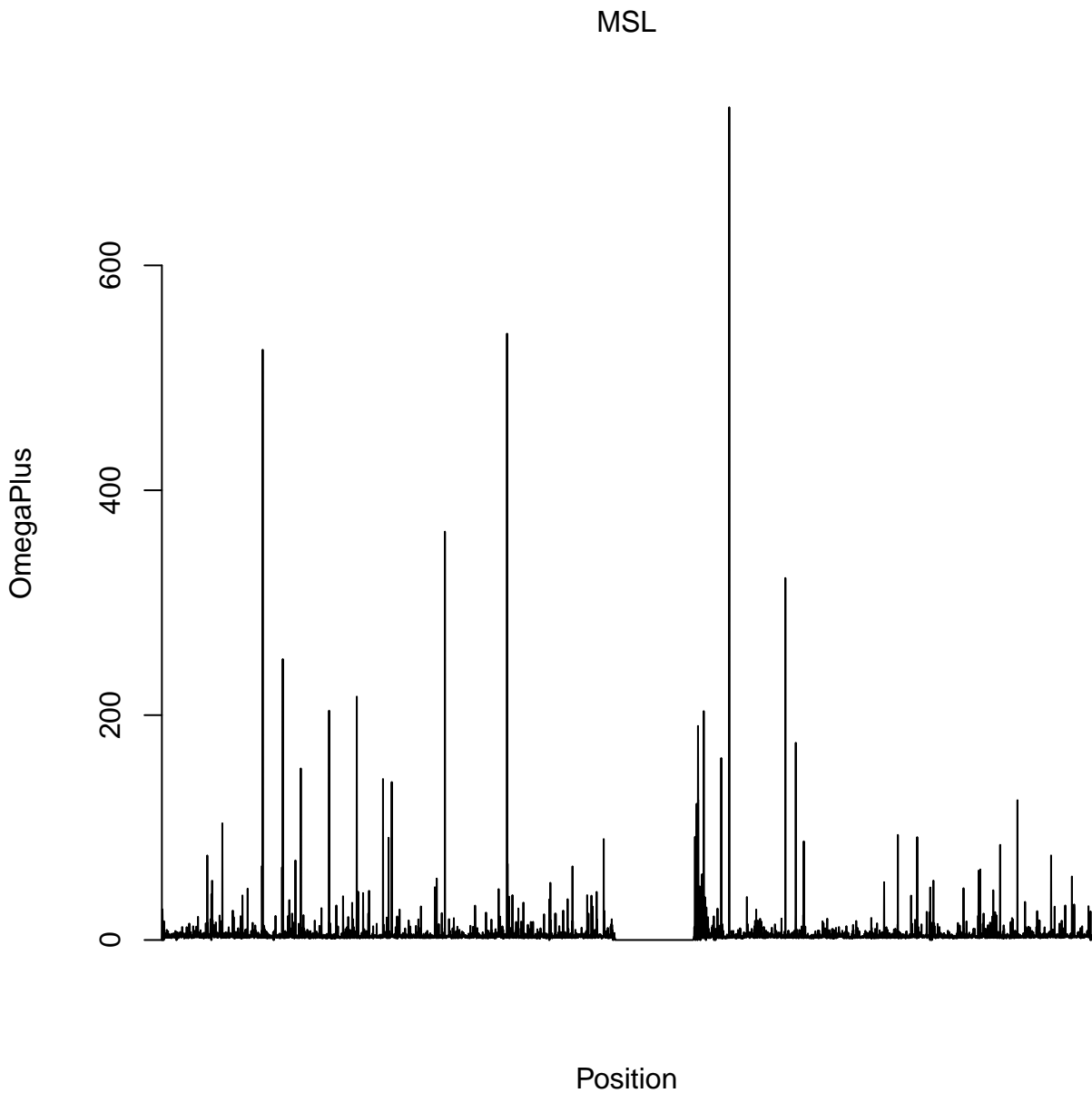

MXL

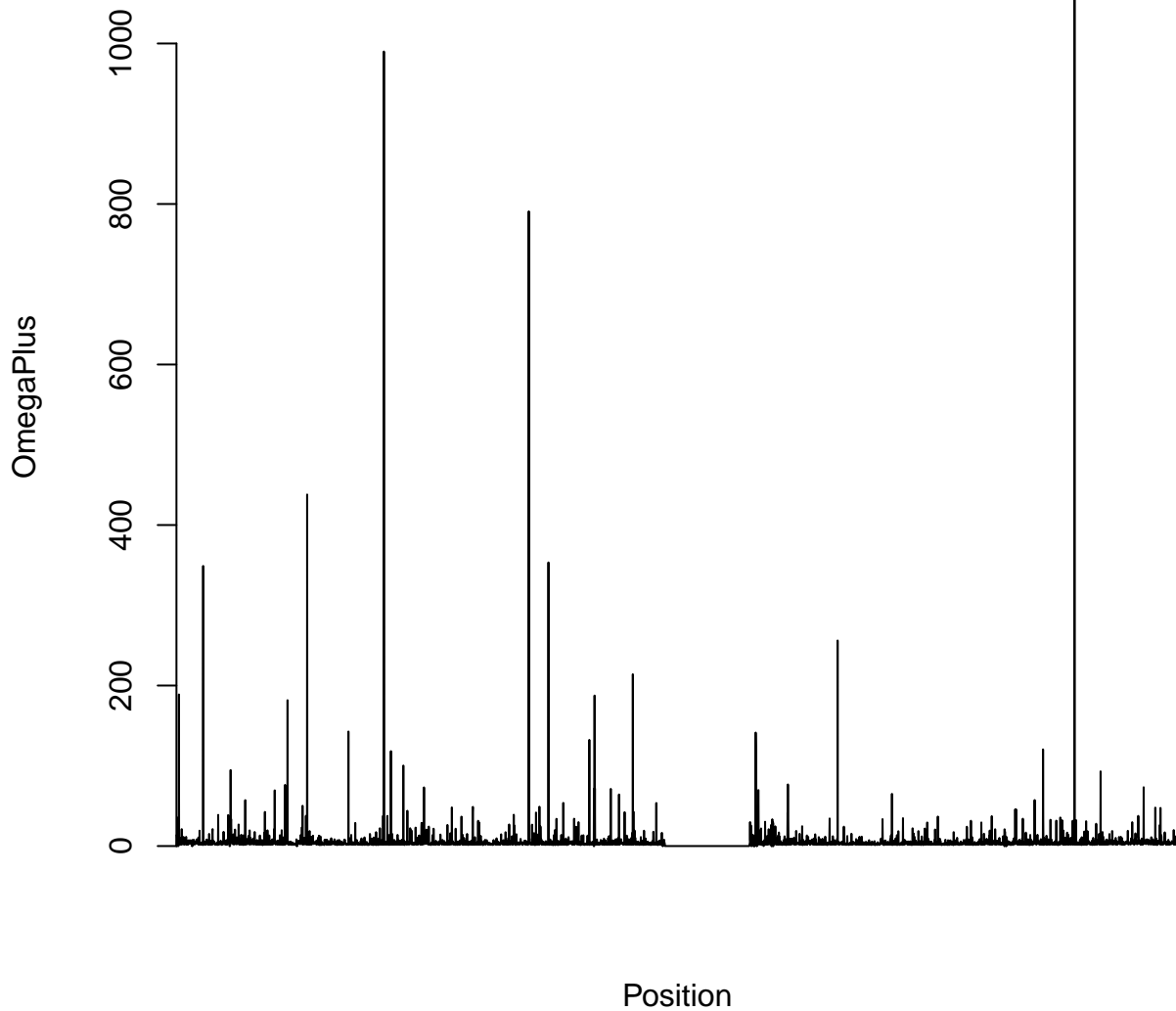

PEL

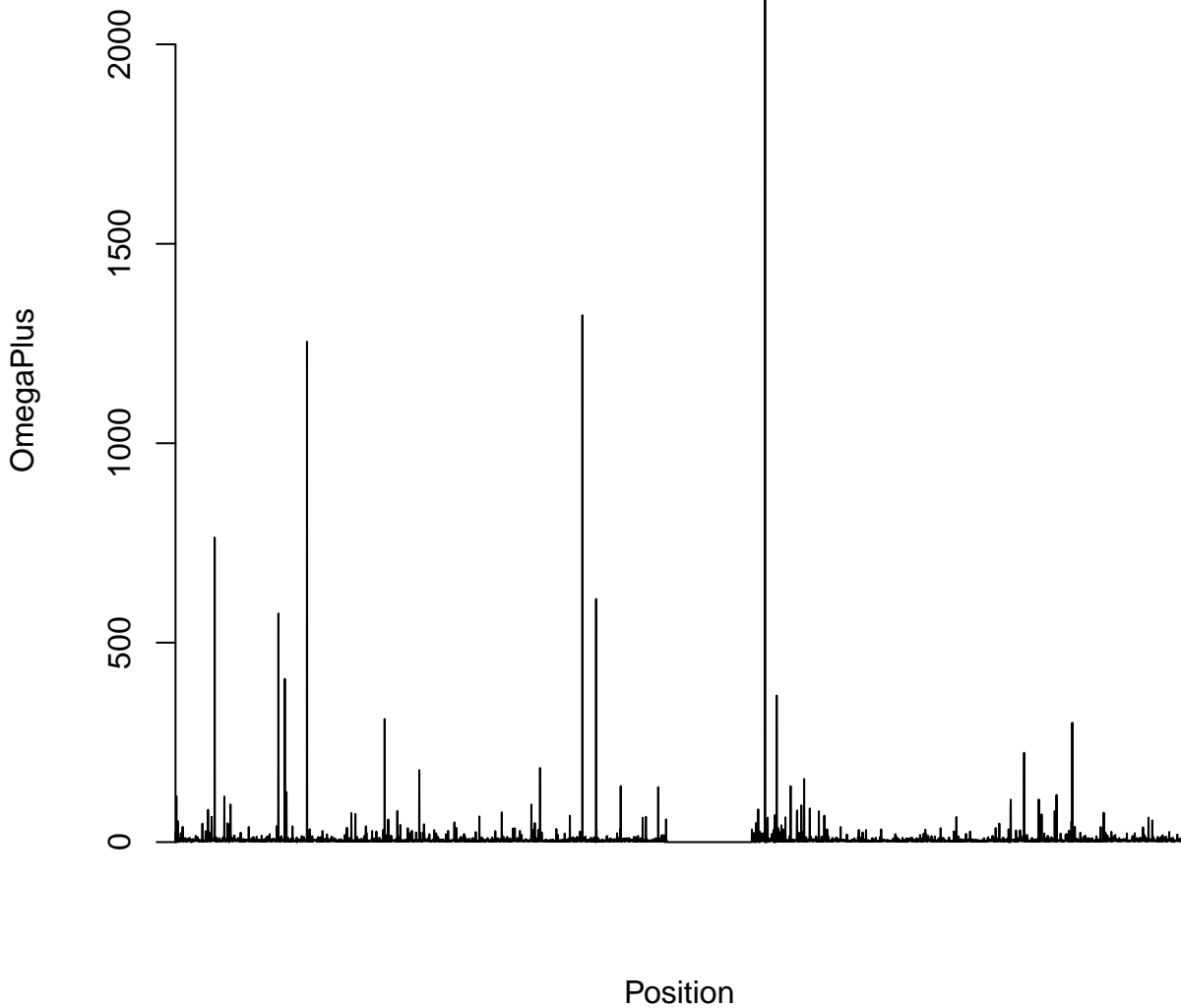

PJL

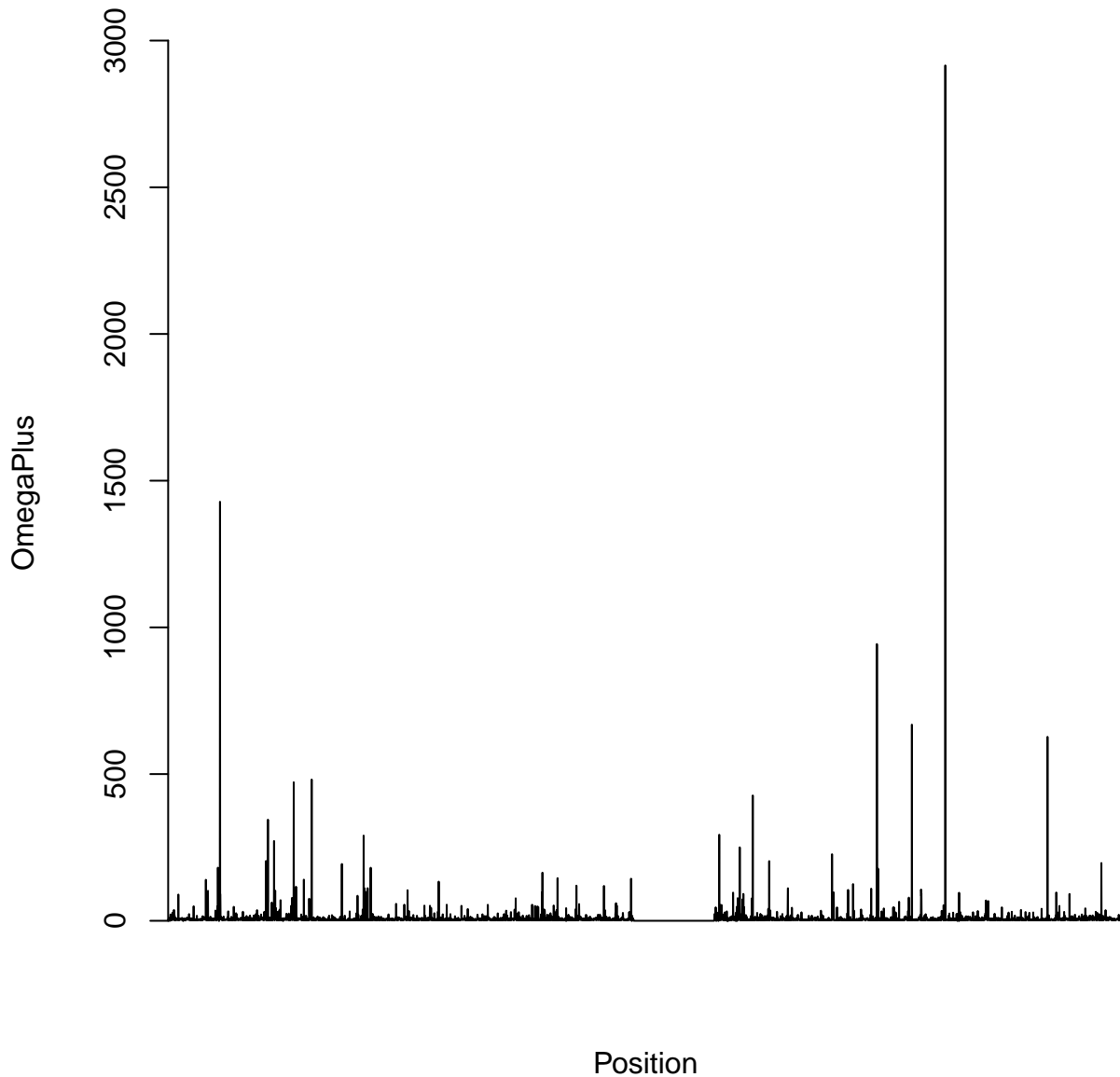

PUR

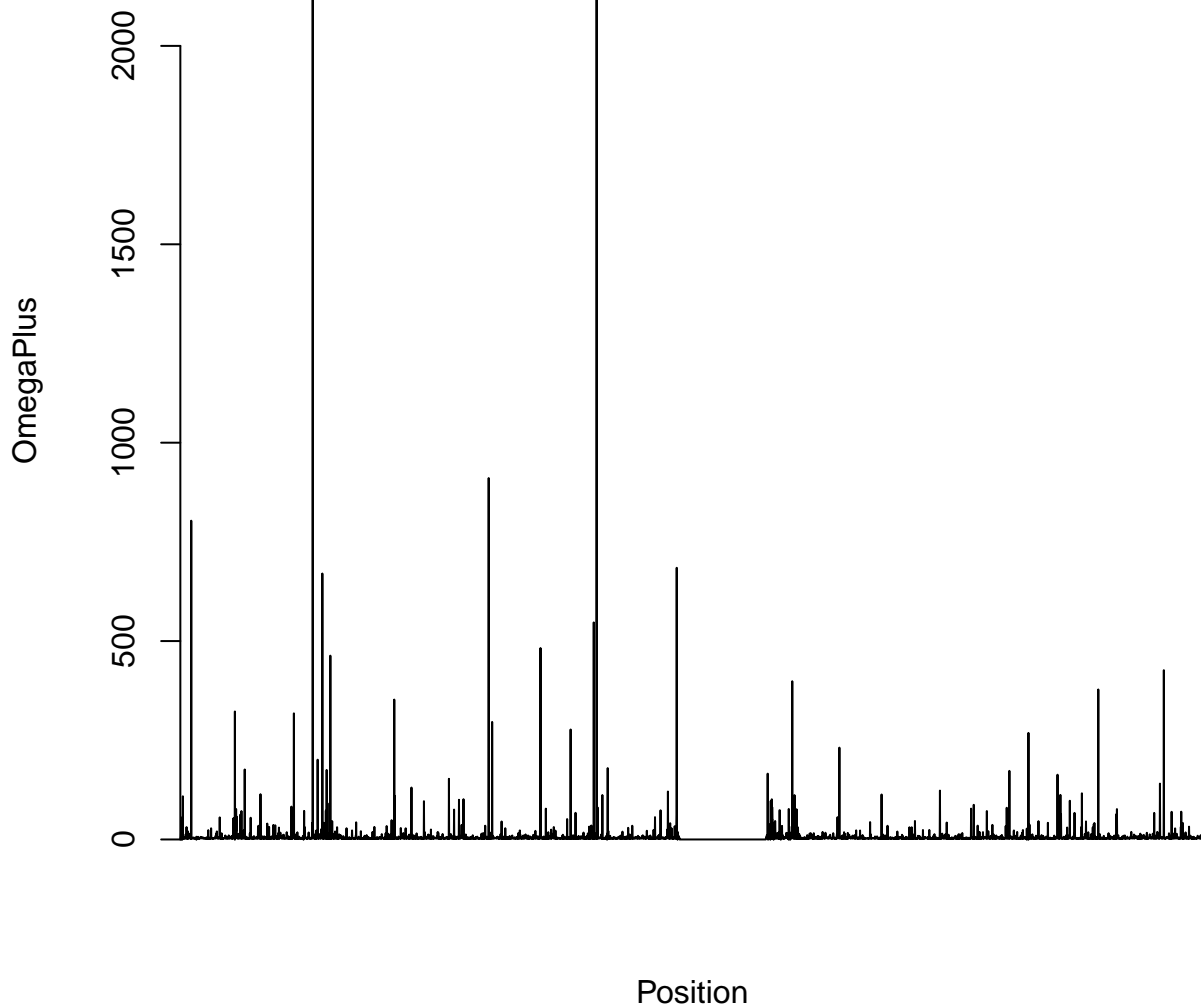

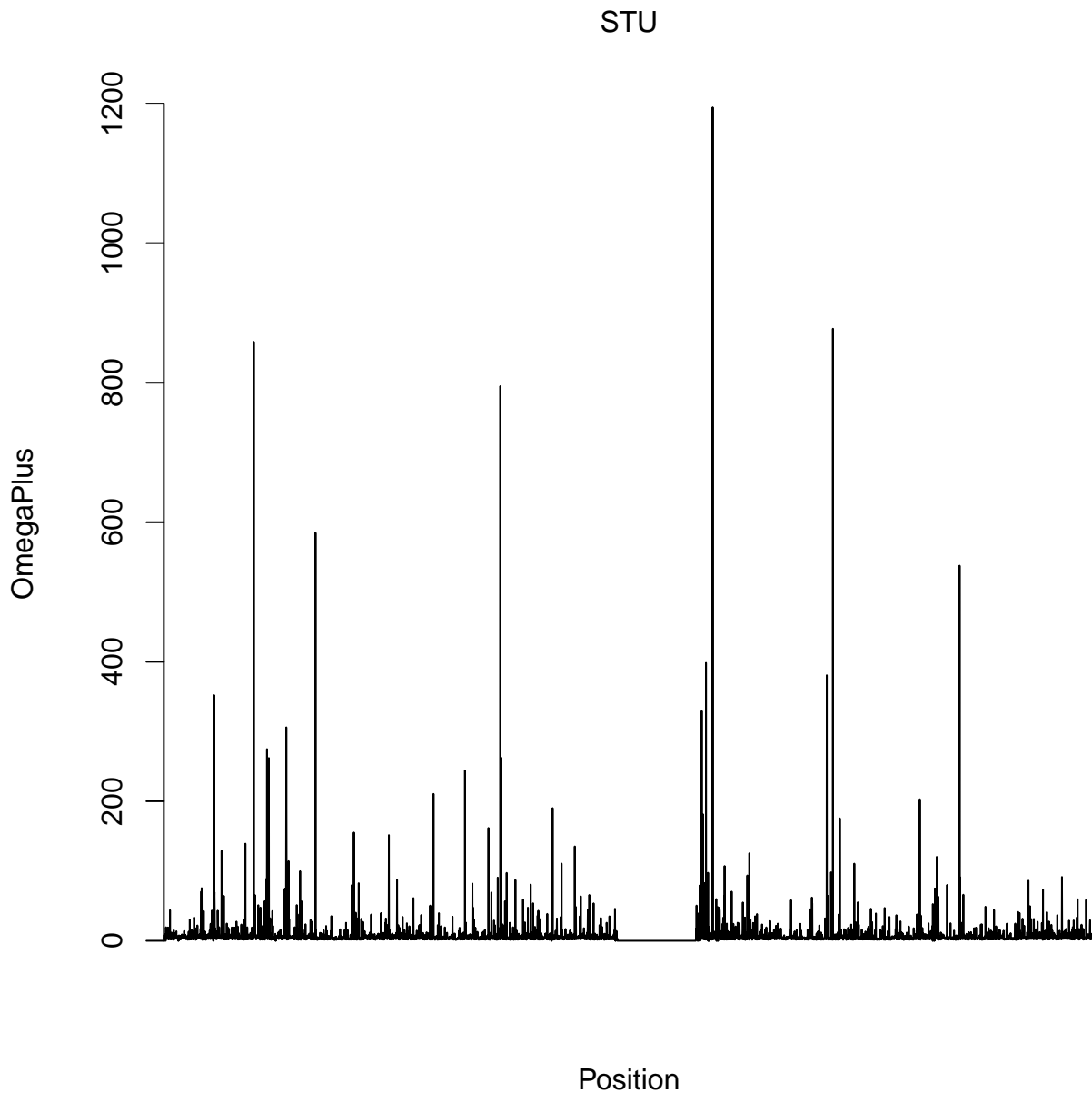

TSI

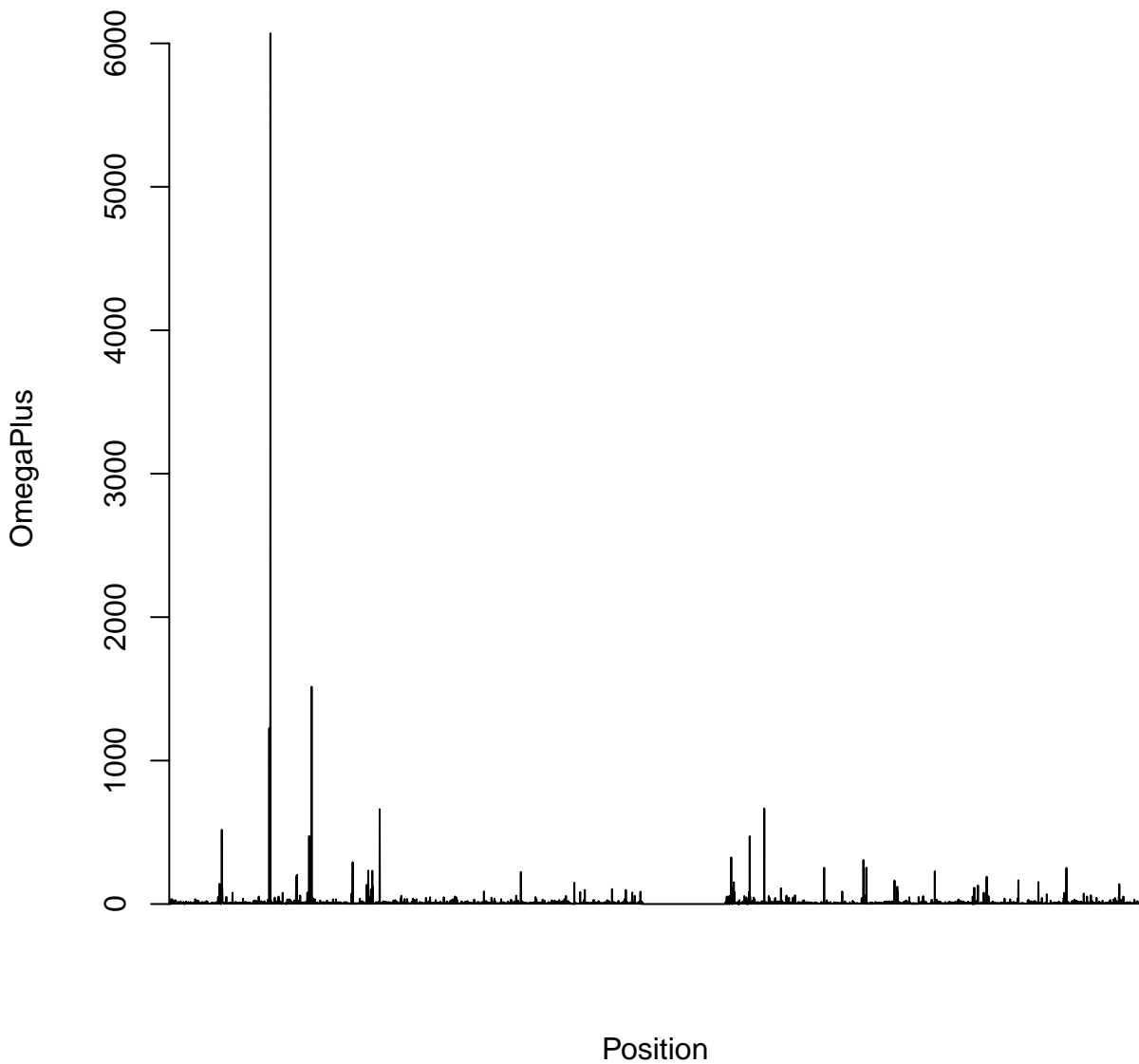

YRI

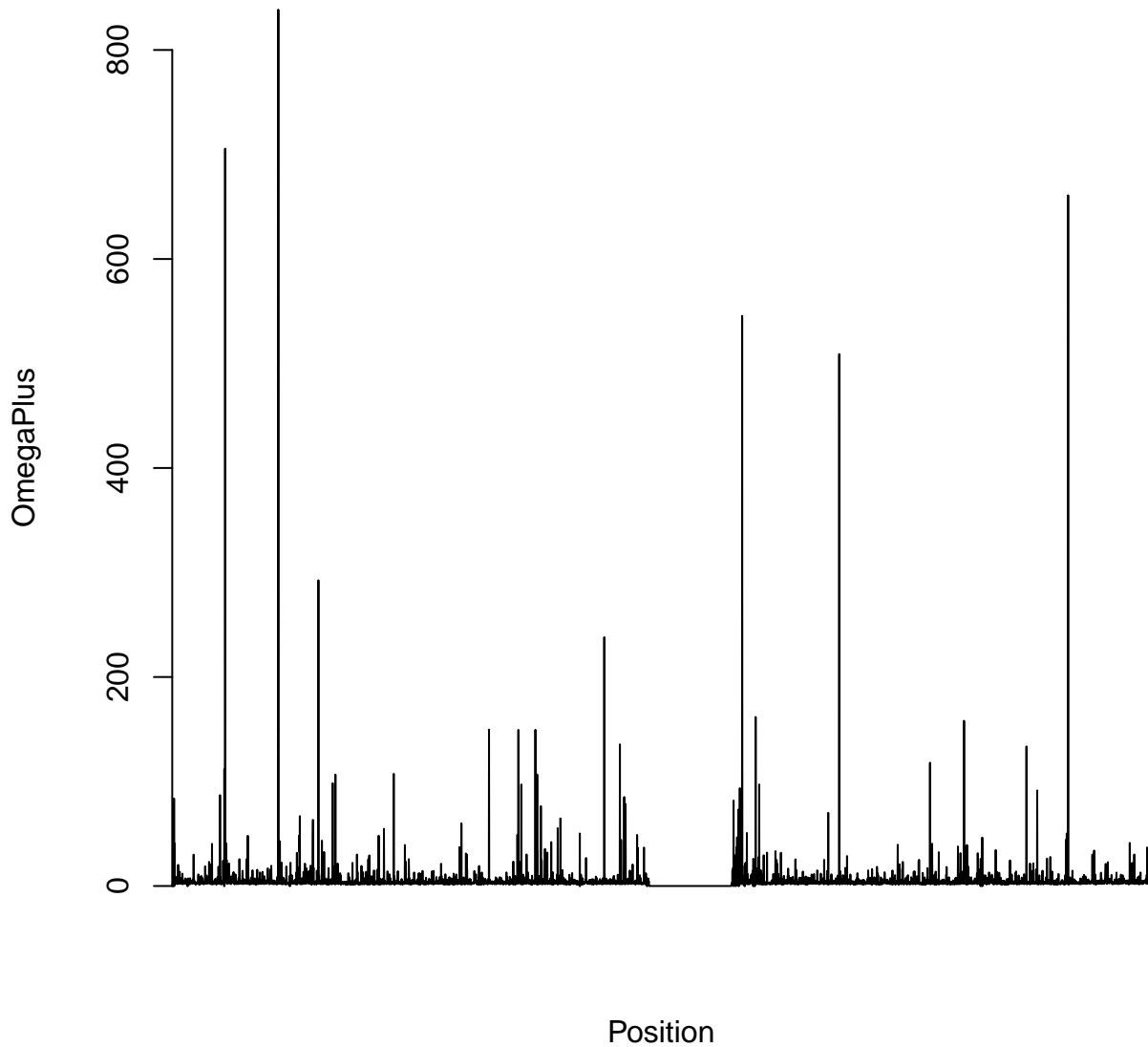

Supplement: Supplementary file 3 — Plots of OmegaPlus scores per population along Human Chromosome 1. This file contains a series of plots (26 plots, one per population) of the OmegaPlus scores along the human chromosome 1. (ZIP 512 kb) [file 13742_2016_114_MOESM3_ESM.zip › additional_file_3/additional_file3.pdf]
