# Supplementary material for: Scalable linkage-disequilibrium-based selective sweep detection: a performance guide
Source: Gigascience. 2016 Feb 8;5:7. doi: 10.1186/s13742-016-0114-9 (PMC4746822; doi:10.1186/s13742-016-0114-9)
Supplement: Supplementary file 60 [file 13742_2016_114_MOESM4_ESM.zip › additional_file_4/AdditionalFile4.odt]

Command lines used for the simulations (Figure 13 and Figure 14)

## Trajectory files

Every trajectory file (e.g. trajectory26.txt, trajectory1.txt, etc) was generated using a forward simulator for the evolution of the beneficial allele and the same demographic model as the one described in the command line of the bottleneck models.

| bottleneck1 | trajdemog -nreps 1000 -t 3200 -s 0.02 -h 0.5 -pfinal 1.0 -eps 1e-3 -eN 20000 25000.0 -eN 20080 50000.0 -npres 50000  -seed 7687 |
| --- | --- |
| bottleneck2 | trajdemog -nreps 1000 -t 3200 -s 0.02 -h 0.5 -pfinal 1.0 -eps 1e-3 -eN 3000 25000.0 -eN 3200 50000.0 -npres 50000  -seed 8855 |
| bottleneck3 | trajdemog -nreps 1000 -t 3200 -s 0.02 -h 0.5 -pfinal 1.0 -eps 1e-3 -eN 3000 500.00 -eN 3400 50000.0 -npres 50000  -seed 18302 |
| bottleneck4 | trajdemog -nreps 1000 -t 3200 -s 0.02 -h 0.5 -pfinal 1.0 -eps 1e-3 -eN 3000 250.000 -eN 3200 50000.0 -npres 50000  -seed 18510 |
| bottleneck5 | trajdemog -nreps 1000 -t 3200 -s 0.02 -h 0.5 -pfinal 1.0 -eps 1e-3 -eN 3000 5000.0 -eN 3400 50000.0 -npres 50000  -seed 30014 |
| bottleneck6 | trajdemog -nreps 1000 -t 3200 -s 0.02 -h 0.5 -pfinal 1.0 -eps 1e-3 -eN 2000 25000.0 -eN 2080 50000.0 -npres 50000  -seed 14394 |
| bottleneck7 | trajdemog -nreps 1000 -t 3200 -s 0.02 -h 0.5 -pfinal 1.0 -eps 1e-3 -eN 2000 5000.0 -eN 2080 50000.0 -npres 50000  -seed 26318 |

The software 'trajdemog' is based on a software from Dick Hudson. It is modified to add the demographic model in the command line. The code is available  from: [http://pop-gen.eu/wordpress/software/trajdemog](http://pop-gen.eu/wordpress/software/trajdemog)

The command lines specify:

-nreps 1000 : number of generated trajectories

-t 3200 : the beneficial allele appeared in the population 3,200 generations ago

-s 0.02 : selection coefficient for the beneficial allele

-h 0.5 : dominance coefficient

-pfinal 1.0 : the final frequency of the beneficial mutation. If a simulation does not reach the final frequency then it is repeated. The value given by the -eps flag specifies the tolerance level regarding the acceptance or rejection of the trajectory.

-eps 0.001 : if the final trajectory frequency is less than 0.999 then reject it

-eN X Y : change the population size to Y, X generations in the past

-npres 50000 : the current effective population size

-seed X : random seed

An example trajectory is show below in Supplementary Figure 4.1

Figure S4.1: An example trajectory (from bottleneck2 model). The beneficial allele appeared in the population about 0.015 x 4N generations in the past. Then its frequency increased and eventually it reached fixation

The command lines for every demographic model used to generate the results shown in Figure 13 and Figure 14 are the following:

## constant model (Hudson's ms):

ms 20 2000 -t 2000 -r 2000 2000

## bottleneck2 model (Hudson's mssel):

mssel 20 1000 0 20 ./trajectory26.txt 1000 -t 2000 -r 2000 2000 -eN .015 0.5 -eN .016 1

## bottleneck3 model (Hudson's mssel):

mssel 20 1000 0 20 ./trajectory33.txt 1000 -t 2000 -r 2000 2000 -eN .015 0.01 -eN .017 1

## bottleneck4 model (Hudson's mssel):

mssel 20 1000 0 20 ./trajectory35.txt 1000 -t 2000 -r 2000 2000 -eN .015 0.005 -eN .016 1

## bottleneck5 model (Hudson's mssel):

mssel 20 1000 0 20 ./trajectory30.txt 1000 -t 2000 -r 2000 2000 -eN .015 0.1 -eN .0171

## bottleneck6 model (Hudson's mssel):

mssel 20 1000 0 20 ./trajectory37.txt 1000 -t 2000 -r 2000 2000 -eN .01 0.5 -eN .0104 1

## bottleneck7 model (Hudson's mssel):

mssel 20 1000 0 20 ./trajectory40.txt 1000 -t 2000 -r 2000 2000 -eN .01 0.1 -eN .0104 1 

## Download link for the R pipelines to combine OmegaPlus with SweeD results

### First R script

[http://pop-gen.eu/wordpress/selective-sweep-analysis-pipelines/combine-sweed-analysis-with-omegaplus-analysis-to-detect-common-outliers](http://pop-gen.eu/wordpress/selective-sweep-analysis-pipelines/combine-sweed-analysis-with-omegaplus-analysis-to-detect-common-outliers)

### Second R script

[http://pop-gen.eu/wordpress/selective-sweep-analysis-pipelines/combine-omegaplus-and-sweed-thresholds-from-simulations](http://pop-gen.eu/wordpress/selective-sweep-analysis-pipelines/combine-omegaplus-and-sweed-thresholds-from-simulations)
